# Advances in the Application of Black Phosphorus-Based Composite Biomedical Materials in the Field of Tissue Engineering

**DOI:** 10.3390/ph17020242

**Published:** 2024-02-13

**Authors:** Wanying Qi, Ru Zhang, Zaishang Wang, Haitao Du, Yiwu Zhao, Bin Shi, Yi Wang, Xin Wang, Ping Wang

**Affiliations:** 1 School of Pharmacy, Shandong University of Traditional Chinese Medicine, Jinan 250355, China; qwysddz@163.com (W.Q.); zhangru793@163.com (R.Z.); 2 School of Pharmacy, Guilin Medical University, Guilin 541001, China; wzs980807@163.com; 3 Shandong Academy of Chinese Medicine, Jinan 250014, China; kkitdht@foxmail.com (H.D.); zywkaka@163.com (Y.Z.); wyi_1989@163.com (Y.W.); 4 Shandong Medicinal Biotechnology Center, Jinan 250062, China; sdyky-shibin@163.com; 5 State Key Laboratory of Biobased Material and Green Papermaking, Faculty of Light Industry, Qilu University of Technology (Shandong Academy of Sciences), Jinan 250353, China

**Keywords:** black phosphorus, two-dimensional nanomaterials, tissue repair, bone defect, skin injury, nerve injury

## Abstract

Black Phosphorus (BP) is a new semiconductor material with excellent biocompatibility, degradability, and optical and electrophysical properties. A growing number of studies show that BP has high potential applications in the biomedical field. This article aims to systematically review the research progress of BP composite medical materials in the field of tissue engineering, mining BP in bone regeneration, skin repair, nerve repair, inflammation, treatment methods, and the application mechanism. Furthermore, the paper discusses the shortcomings and future recommendations related to the development of BP. These shortcomings include stability, photothermal conversion capacity, preparation process, and other related issues. However, despite these challenges, the utilization of BP-based medical materials holds immense promise in revolutionizing the field of tissue repair.

## 1. Introduction

The repair and regeneration of tissue and organ injuries are crucial for clinical disease treatment. Currently, in clinical practice, four strategies are commonly employed: surgical repair, autograft transplantation, allograft transplantation, and the use of artificial products. However, these strategies face challenges such as limited sources, immune rejection, and poor biocompatibility. Black phosphorus (BP) is a two-dimensional direct bandgap semiconductor nanomaterial known for its excellent biological effects, degradability, and optical and electrical properties, making it a subject of wide interest in the field of biomedical research. In recent years, the construction of composite biomedical materials based on black phosphorus and their application in tissue engineering have attracted significant attention, demonstrating promising prospects for clinical transformation.

This paper reviews the physicochemical properties and range of applications of BP material. The shortcomings of the material are also discussed. Through our summary, we hope to provide a theoretical basis for the further application of BP in biomedicine.

## 2. The Physicochemical Properties and Biological Effects of Black Phosphorus Materials

**Figure 1:**
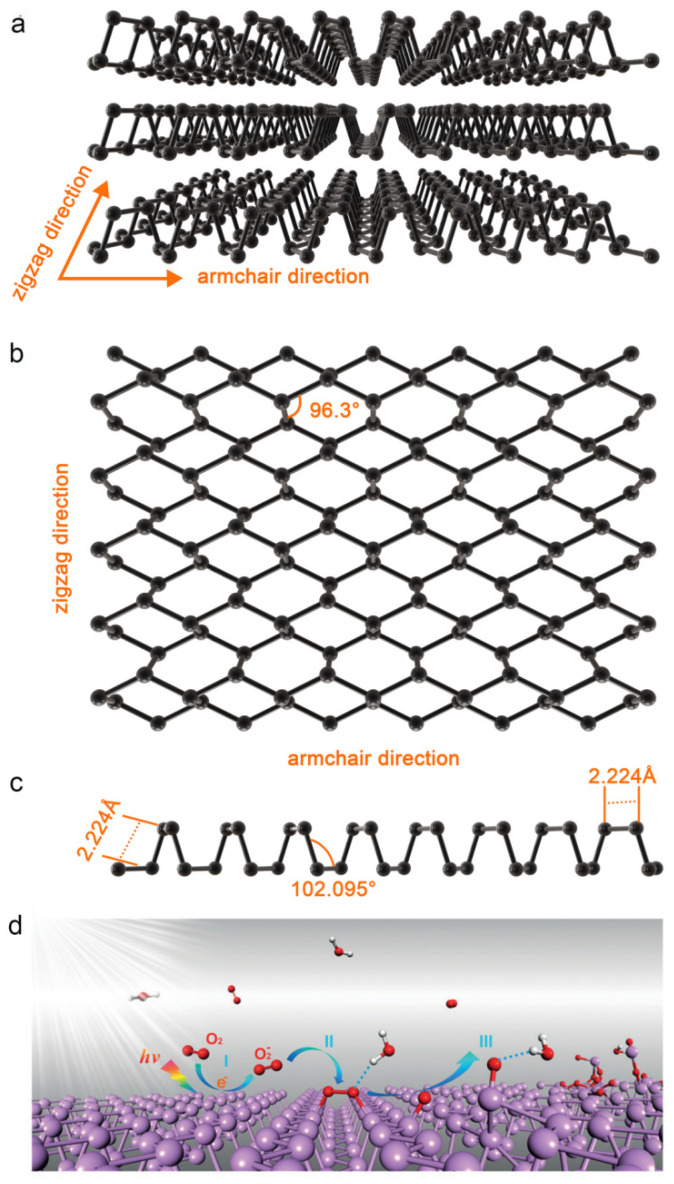
BP crystal structure images: (**a**) 3D view; (**b**) top view; (**c**) side view; (**d**) BP produces ROS [7].

Black phosphorus (BP) is a single-element, two-dimensional crystal that is black in color and exhibits a metallic luster. It is an allotrope of phosphorus that can be formed from red phosphorus or white phosphorus under high-temperature and high-pressure conditions [1] and can exist stably in nature [2,3,4]. The bulk BP possesses a unique layered structure, in which each phosphorus atom is linked by covalent bonds to form a single layer, and the adjacent atomic layers are stacked together by weak van der Waals force interactions [5], presenting a folded non-planar hexagonal ring structure with a relatively large surface area. BP’s crystal structure exhibits folding in the armchair direction and a zigzag direction, leading to differences in covalent bond lengths and bond angles in these two directions (Figure 1a–c). BP’s in-plane anisotropic structure gives rise to its unique optical, mechanical, and electrical properties [6], making it of significant interest in the field of biomedical research.

The construction of BP biomedical composite materials is primarily based on nanoscale single-layer black phosphorus nanosheets (BPNS) [8] and even smaller black phosphorus quantum dots (BPQDs) [4]. Currently, the preparation methods for BP nanomaterials mainly include mechanical exfoliation, liquid-phase exfoliation, chemical vapor deposition, and pulsed laser deposition methods [8]. The more mature preparation methods of BP nanomaterials mainly include the “top-down” and “bottom-up” methods. The “top-down” method means breaking the force of BP layer and layer through molecular insertion or mechanical force and realizing the BP stripping of block BP into a single layer or less layer, such as the mechanical stripping method, electrochemical stripping method, and liquid phase stripping method; the “bottom-up” method means assembling the smaller structural unit into a large structural system, such as vapor deposition method and solvent thermal method, through gas phase and vacuum evaporation. At present, new preparation methods such as pulsed laser deposition method are gradually emerging, providing a brand new preparation direction for the preparation of BP. Since the first isolation of BPNS in 2014 [9], BP has found widespread applications in fields such as batteries, field-effect transistors, and electronics, owing to its unique wrinkled structure, tunable direct bandgap, high carrier mobility, and in-plane anisotropy [10]. As research has progressed, BP’s excellent photothermal effects, photodynamic effects, biocompatibility, drug delivery capabilities, and osteoinductive properties have gradually emerged, garnering increasing attention in tissue engineering and nanomedicine.

### 2.1. Physical and Chemical Properties

#### 2.1.1. Photothermal Effects

The bandgap related to the number of BP layers fluctuates within the range of 0.3 (bulk) to 2.0 (layers) eV [11,12] (Figure S1), covering the spectral region from visible light to mid-infrared [13,14]. It exhibits high photothermal conversion efficiency in the near-infrared (NIR) region [15,16], making it an excellent candidate for photothermal therapy (PTT) [17]. For instance, BP generates localized high temperatures under induction, leading to the upregulation of bone cell regulatory factors such as alkaline phosphatase (ALP) [1] and heat shock proteins (HSP), thus accelerating the bone repair process. Furthermore, elevated temperatures cause protein denaturation within cells, which can offer broad applications in the fields of infection control and oncology [18,19]. Additionally, BP can enhance blood–brain barrier permeability through PTT, successfully crossing the blood–brain barrier and selectively capturing Cu^2+^ to protect neuronal cells, holding significant potential for central nervous system disease treatment [20]. Jin et al. [21] designed a BPNS-fluoxetine delivery platform which, under NIR irradiation, significantly alleviated mouse depression-like behavior by increasing the expression of hippocampal neurotrophic factors, reducing the excitability of amygdala-projecting neurons, and lowering the frequency of miniature excitatory postsynaptic currents. Xiong et al. [22] constructed a nano-complex of BP loaded with paeoniflorin and brain-targeting ligand transferrin, which traversed the blood–brain barrier through endocytosis in brain capillary endothelial cells for targeted treatment of Parkinson’s syndrome.

#### 2.1.2. Photodynamic Effects

BP can be used as a photosensitizer for photodynamic therapy (PDT) [23,24]. After exposure to light, BP converts the light energy into molecular oxygen, which is then motivated and transformed into highly reactive oxygen species (ROS) [25], such as hydroxyl radicals (∙OH) and singlet oxygen (^1^O_2_), which inhibit cell proliferation and induce apoptosis through the oxidative action of ROS [26]. The generation of ROS can be divided into three steps: Step I: O_2_^−^ is generated through a charge transfer reaction under ambient light (O_2_ + *hv* $\overset{p}{\to }$ O_2_^−^ + h^+^; P and h^+^ stand for phosphorene and a hole, respectively); step II: O_2_^−^ dissociates at the surface and forms two P-O bonds with the phosphorene (O_2_^−^ + P + h^+^ → P_x_O_y_); step III: through the hydrogen–bond interaction, water molecules draw the bonded O and remove P from the surface and break the top layer of phosphorene. (Figure 1d) [7]. Studies have shown that BP achieves sterilization rates of up to 91.65% for *Escherichia coli* (*E. coli*) and 99.69% for *Bacillus subtilis* (BS) [27] without inducing antibiotic resistance, thus exhibiting a broad-spectrum antibacterial effect [28]. Compared with non-metallic PDT materials such as graphene and silicon, BP is a semiconductor with a thickness-dependent bandgap, which can be varied from about 0.3 eV for bulk to 2.0 eV for a single layer, indicating the broad absorption across the ultraviolet and entire visible light region, so BP has a higher ROS generation rate (9.1%) [29], providing a significant advantage in anti-infection and antibiotic resistance.

#### 2.1.3. Drug Loading Performances

The electronegativity and large surface area of BP make it an excellent drug carrier. BP surfaces carry a negative charge, allowing them to bind with positively charged drugs [30]. Its unique armchair-shaped structure provides BP with a larger surface area compared to typical 2D materials, enhancing drug-loading capacity. BPNS achieves a high doxorubicin loading rate of up to 950% [31], coupled with its outstanding heat-responsive capability, enabling controlled drug release via light modulation [32]. This establishes an efficient and precise drug delivery platform. However, BP degrades relatively quickly in physiological environments, diminishing therapeutic efficacy. Hence, researchers have improved BP’s stability and photothermal conversion efficiency through surface modifications using materials such as red blood cell membranes [33,34], polyethylene glycol [1], and polydopamine [35].

#### 2.1.4. Electrical Conductivity

Due to its high charge carrier mobility (10^4^ cm^2^/V·s) and pronounced anisotropy, BP exhibits excellent conductivity and is widely used in fields such as optoelectronics, biomedicine, batteries, transistors, and electronic components [36]. Research has revealed the distinct advantages of conductive materials in the repair of cardiac and peripheral nervous tissues. For instance, graphene nanofiber scaffolds fabricated by Wang et al. have been shown to promote Schwann cell migration, proliferation, myelination, secretion of neurotrophic factors, and specific gene expression related to myelination [37]. Similarly, carbon nanotube scaffolds have been observed to facilitate the differentiation and proliferation of neural progenitor PC12 cells [38]. Given its superior electrical conductivity compared to other 2D materials, BP holds immense potential in the fields of restoring damaged neurons or cardiac cells and promoting neural or cardiac regeneration.

### 2.2. Biological Characteristics

#### 2.2.1. Biocompatibility and Degradability

Based on current research findings, BP materials exhibit promising biocompatibility, but the unreasonable use of BP can also cause potential harm to the body. Firstly, phosphorus, which constitutes BP, is one of the elements with higher concentrations in the human body, accounting for 1% of the total body mass (85% of phosphorus is found in bones and teeth, serving as an essential element for maintaining skeletal mechanical strength and inducing bone regeneration) [39]. Secondly, phosphorus serves as a major component of genetic materials such as nucleic acids, contributing significantly to vital life processes, nerve signal transmission, and catalytic reactions [40,41]. However, as research advances, it has been observed that an excessive amount of BP can also lead to harm to the organism [42]. Therefore, precise control over the dosage of BP materials is essential in its applications.

Due to its susceptibility to oxidation, BP possesses degradability. When BP comes into contact with oxygen, it readily forms superoxide ions (O_2_^•−^), which subsequently transform into harmless phosphate ions in the human body [43]. BP undergoes rapid degradation and is efficiently cleared from the body, thereby reducing subsequent adverse reactions, which is a significant advantage of its in vivo use. The degradation rate of BP is jointly regulated by oxygen, water, and light and is inversely correlated with its thickness; thinner BP degrades more rapidly [7]. Compared to other two-dimensional nanomaterials such as molybdenum disulfide, boron nitride, and graphene, BP’s biodegradability avoids the accumulation of foreign substances in both intra and extracellular environments, reducing cellular toxicity and enhancing biocompatibility, thus demonstrating unique advantages in the field of tissue repair.

#### 2.2.2. Osteoinduction

**Figure 2:**
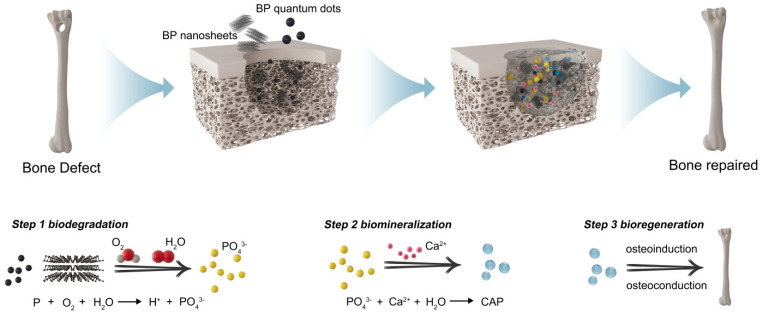
Mechanisms of bone formation promoted by BP.

BP is composed of a single phosphorus element with a high degree of homology to the inorganic components of natural bone. Furthermore, BP readily adsorbs surrounding oxygen molecules, undergoing oxidation to form phosphate, followed by the adsorption of free calcium ions to create calcium phosphate, thereby promoting in situ bone repair and mineral deposition [44] (Figure 2). Additionally, BP possesses strong photothermal conversion capabilities in the NIR range, enabling localized hyperthermia to upregulate the expression of bone-forming factors such as ALP and HSP, enhancing mineralization capacity and thereby accelerating bone tissue repair [45]. Consequently, BP has significant potential in the field of bone injury repair.

#### 2.2.3. Antibacterial Properties

BP shows its superior bactericidal ability. At present, the antibacterial mechanism and application of BP can be divided into the following categories:

(a) Physical damage caused by membrane damage. BP has an orthogonal crystal structure and fold surface, which can effectively make contact with the bacterial surface. The use of rough surfaces and sharp edges to destroy the bacterial membrane, causing bacterial death, is one of the mechanisms underlying the antimicrobial activity of BP, also known as the nanoknife mechanism [46]. BP exhibits time-and concentration-dependent antimicrobial activity against both Gram-negative and -positive bacteria, as the sharp edges of BPNS in the interaction with bacteria can cause physical damage to the bacterial membrane and RNA leakage, resulting in bacterial death [47].
(b) ROS generation. Oxidative stress caused by ROS is the main mechanism of bacterial death, and ROS kills bacterial pathogens by destroying the cell membrane and bacterial intracellular molecules such as DNA, RNA, and protein interactions. Shaw demonstrated that the bactericidal properties of BP arise from its unique ability to produce ROS and have excellent antimicrobial effects against sensitive and resistant bacteria and even fungi [48]. BPNS can be used as an effective photosensitizer to produce ^1^O_2_ with a quantum yield of about 0.91. The bactericidal rate of BPNs to *E. coli* and *B. subtilis* reached 91.65% and 99.69%, proving that oxidative stress caused by ROS is one of the bactericidal mechanisms [46]. Tan et al. prepared antibiofilm containing BP and produced ^1^O_2_ under visible light to 99.3% and 99.2% [49].
(c) The photothermal effect destroys the bacteria. The photothermal effect can inhibit bacterial growth and kill bacteria by altering bacterial cell membrane permeability and signal transmission pathways. BP has high photothermal conversion efficiency under near-infrared light sources [50]. Zhang et al. improved the stability of BP with quaternary chitosan, showing excellent photothermal capacity. The temperature of BP increased by nearly 30 °C after 10 min at 808 nm, and the sterilization rate against methicillin *S. aureus* and *E. coli* was greater than 95% at low doses [51].

#### 2.2.4. Promotion of Neuronal Differentiation

BP exhibits unique biological effects on stem cells, enhancing the survival and neural differentiation of neural progenitor cells (NPCs) through two pathways: inducing the nuclear factor E2-like 2 (Nrf2) signaling pathway and regulating the oxidative-reductive homeostasis within stem cells. This results in robust neuroprotection and axon regeneration capacity in NPCs. Moreover, when employed as a blank nanocarrier in stem cell therapy, BP demonstrates promising therapeutic outcomes. In the future, the assembly of BP with gene carriers, growth factors, and small molecule drugs holds the potential to become a powerful tool for improving stem cell survival and inducing neuronal differentiation, thereby offering novel insights into the treatment of neurological disorders [52].

### 2.3. Potential Clinical Applications

In the field of traditional Chinese medicine (TCM), the application of BP composite biomaterials is still in its nascent stage. BP exhibits significant potential as an exceptional carrier for loading active components of TCM due to its high drug-loading capacity, excellent photothermal conversion efficiency, and strong targeting capabilities. Zhao [53] utilized BPQDs to load Jolkinolide B (JB), which not only enhanced the pharmacokinetics of JB but also promoted the apoptosis rate of Raji cells by increasing the generation of intracellular ROS. He et al. [54] developed a photothermal-responsive ‘chanterelle-BPNS hydrogel system,’ enabling the on-demand release of chanterelle and simultaneously reducing its cardiotoxicity, thus showing enormous potential in synergistic thermotherapy for tumors. Huang et al. [55] employed sialic acid-modified BPNS to load sesquiterpenoids compound, isolinderalactone, for the treatment of *Helicobacter pylori*-infected mice, which yielded more significant effects than conventional triple therapy, offering a novel strategy for *Helicobacter pylori* treatment. Consequently, the establishment of BP composite biomaterials with TCM active ingredients as a matrix or the utilization of BP composite biomaterials as a platform for intelligent drug delivery of TCM formulations holds paramount significance in advancing TCM activity and the development of novel pharmaceutical formulations.

## 3. BP-Based Composite Biomaterials for Tissue Repair

BP possesses a multitude of advantages, including photothermal and photodynamic effects, drug-loading capability, conductivity, biocompatibility, degradation properties, bone-inducing potential, and antibacterial properties. It has garnered increasing attention and achieved significant research outcomes in the field of tissue engineering. However, the standalone use of BP presents challenges such as susceptibility to degradation and relatively poor mechanical properties, which impede the progress of tissue repair. To better simulate the microenvironment of natural tissues, the development of multifunctional BP composite medical materials is considered an effective approach. In recent years, a wide range of BP composite biomaterials for tissue engineering has emerged, spanning various areas such as bone repair and regeneration, skin repair, and neural vascular repair. These materials take various forms, including hydrogels, scaffolds, electrospun fiber membranes, microspheres, vesicles, and microneedles. This article categorizes BP composite biomaterials according to the aforementioned classifications.

### 3.1. BP-Based Hydrogels

Hydrogels possess a hydrophilic 3D network structure with a water content that can exceed 90% [56,57]. This characteristic facilitates the exchange of oxygen and substances [58], thereby promoting cell implantation, adhesion, and growth [59]. Due to their high biocompatibility, low immunogenicity, and tunable physicochemical properties [60], hydrogels have witnessed unprecedented development in the fields of tissue engineering [61,62]. Consequently, the construction of BP hydrogel composite biomedical materials and their applications in the field of tissue engineering has garnered widespread attention. Based on the application method of BP hydrogel materials, they can be categorized into implantable hydrogels, injectable hydrogels, dressings, and sprayable hydrogels (Table 1).

#### 3.1.1. Implantable BP-Based Hydrogels

**Figure 3:**
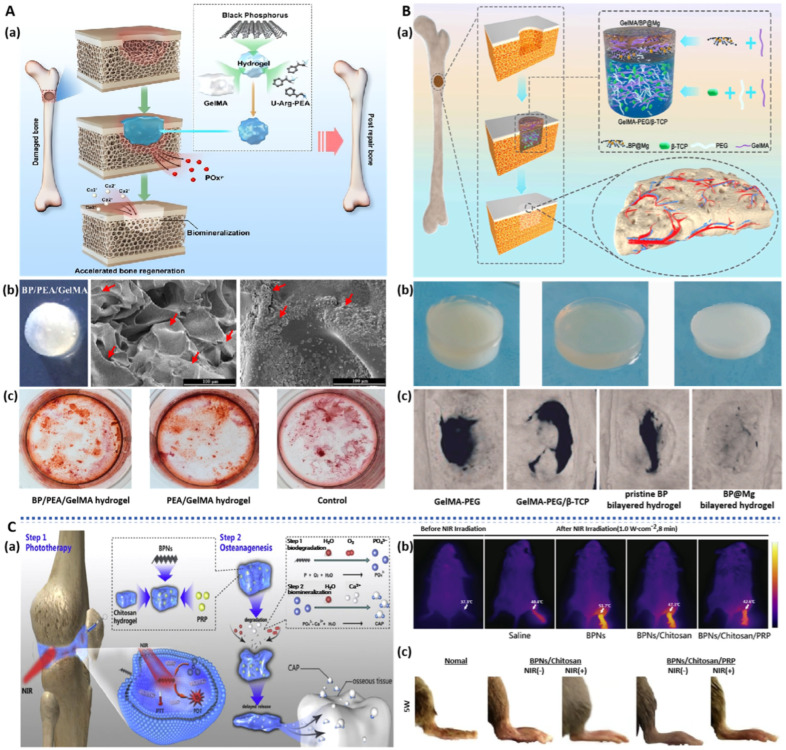
BP-based hydrogels for bone regeneration: (**A**): (**a**) Schematic illustration of BP-based hydrogels for bone regeneration. (**b**) Morphology and SEM images of BP hydrogels after 15 days of mineralization. (**c**) ARS staining images of hDPSCs cultured for 15 days [44]. (**B**): (**a**) Schematic illustration of BP-based hydrogels for bone regeneration. (**b**) Morphology image of BP-based hydrogels (bilayer, upper hydrogel, bottom. (**c**) Micro-CT images of the cranial defect after different treatments for 12 weeks [67]. (**C**): (**a**) Schematic illustration of BP-based hydrogels for bone regeneration. (**b**) In vivo representative photothermal images of arthritis sites after different conditions. (**c**) Lateral and anterior images of affected joints of CIA models after the treatment for 5 weeks [72].

Hydrogels can simulate natural tissue environments and provide structural support to defect sites. BP possesses the ability to induce mineralization of bone tissue, making BP-based hydrogels advantageous for bone repair applications. Huang et al. [44] prepared a BP nanosheet/polyester amide/gelatin methacrylamide (BPNS/PEA/GelMA) hydrogels using ultraviolet (UV) photopolymerization (Figure 3A). Due to the sustained release of phosphate ions by BPNS, this hydrogel can expedite the in situ mineralization deposition of bone cells and induce osteogenic differentiation of human dental pulp stem cells (hDPSCs) through the bone morphogenic protein–runt-related transcription factor 2 (BMP-RUNX2) pathway. Upon implantation in a rabbit cranial bone defect model, this hydrogel accelerates the bone regeneration process, achieving complete healing of the defect site within just 12 weeks.

Implant infection is a common complication of bone transplantation surgery, causing significant suffering and financial burden to patients. Due to its dual attributes of photothermal antibacterial properties and bone-inducing capabilities, BP can be utilized in the treatment of infectious bone defects. Qing et al. [63] prepared BP nanosheet/magnesium oxide/polyvinyl alcohol/chitosan (BPNS/MgO/PVA/CS) hydrogels through interactions such as hydrogen bonding. Leveraging the intrinsic antibacterial properties of CS and the photothermal antibacterial action of BPNS, this hydrogel exhibited antibacterial rates exceeding 99.9% against both *S. aureus* and *E. coli*. Furthermore, the sustained release of Mg^2+^ and PO_4_^3−^ from the gel promoted the migration and osteogenic differentiation of bone marrow mesenchymal stem cells (BMSCs) by activating the Phosphatidylinositide 3 kinases–serine/threonine kinase (PI3K-AKT) signaling pathway, effectively enhancing the repair of rat cranial bone defects. Miao et al. [64] employed UV cross-linking to prepare BPNS/gel hydrogels. The introduction of BPNS enhanced the mechanical strength of the hydrogel and improved its mineralization capacity, significantly increasing bone volume and bone mineral density at the site of rat cranial defects. Additionally, under NIR irradiation, BPNS/gel hydrogels exhibited highly efficient photothermal conversion characteristics, demonstrating remarkable killing efficiency against both *S. aureus* and osteosarcoma cells (Saos-2).

The process of vascular formation is highly coupled with skeletal reconstruction; hence, the designs of hydrogel materials that are both pro-angiogenic and osteoconductive have a promising application in promoting bone injury repair. Xu et al. [65] prepared a hydrogel by crosslinking BPNS, deferoxamine (DFO), and gel solution using UV radiation. BPNS was employed to facilitate the controlled release of PO_4_^3−^ and DFO, activating critical angiogenic genes such as vascular endothelial growth factor (VEGF), thereby promoting angiogenesis and accelerating the process of bone defect repair. In vivo studies confirmed the presence of newly formed bone rich in blood vessels and collagen at the site of tibial defects in rats with acute femoral artery occlusion, and bone regeneration was promoted through the activation of the BMP/Runx_2_ pathway.

Hydrogels not only possess exceptional loading capacity but also offer flexible control over their network topological structure, thus simulating the extracellular matrix microenvironment to promote bone regeneration. Wang et al. [66] employed a combinatorial screening approach to fabricate double-network hydrogels with ultra-high mechanical strength. Due to the synergistic effects of covalent crosslinking and chain entanglement, the compression strength of these double-network hydrogels reached 0.1~15 MPa, facilitating the spreading and osteogenic differentiation of human bone marrow mesenchymal stem cells (hBMSCs) and mouse embryonic osteoblasts (MC3T3). The introduction of BPNS into these double-network hydrogels induced the formation of CaP crystals, thereby enhancing their mineralization capacity and promoting hBMSCs proliferation and adhesion, ultimately facilitating the repair of cranial bone defects, layered connective tissue, and major blood vessels. Xu et al. [67] prepared a double-layered BP-based hydrogel (top layer: GelMA/BPNS/Mg, bottom layer: GelMA/polyethylene glycol/β-tricalcium phosphate) (Figure 3B). Due to BP’s capability to promote angiogenesis and neurogenesis and the addition of Mg ions to enhance its stability, the top hydrogel-induced migration of human umbilical vein endothelial cells (HUVECs) and upregulation of neuro-related proteins in neural stem cells (NSCs), significantly promoting vascular and neural generation. The bottom hydrogel improved the activity and osteogenic differentiation ability of BMSCs. Through the synergistic effect of the double-layer structure, this hydrogel not only enhanced early vascularization and neurogenesis in bilateral cranial bone defects in rats but also accelerated bone regeneration and remodeling.

#### 3.1.2. Injectable BP-Based Hydrogels

The irregular shapes of tissue defects often necessitate minimally invasive repair methods, and the injection of hydrogels as a repair material serves this purpose well. This approach not only avoids the trauma, infections, and scarring associated with surgical implantation but also offers excellent adaptability to irregular defect shapes [68]. Liu et al. [69] introduced electrically conductive carbon nanotubes (CNT) and BPNS into oligo(poly(ethylene glycol) fumarate) (OPF) hydrogels. The precursor solution of BPNS/CNT/OPF hydrogel was injected into irregular defects in rabbit femurs, vertebrae, and lateral spinal columns. It rapidly filled the irregular defect sites and formed hydrogels in situ within the body. The doped BPNS provided a continuous supply of phosphate ions and, under electrical stimulation, upregulated the expression of key osteogenic genes, synergistically promoting the proliferation and differentiation of pre-osteoblast cells, thereby accelerating bone regeneration. Injectable BP hydrogels have also demonstrated significant application value in the treatment of osteoarthritis. Polypyrrole (PPy) is a conductive polymer and provides a microenvironment that mimics neural tissue. Lu et al. [70] developed a biodegradable, conductive, and printable hydrogel based on collagen and a block copolymer of polypyrrole and polycaprolactone (PPy-b-PCL) for bioprinting of neural tissue constructs. The collagen/PPy-b-PCL hydrogels possessed better printability and biocompatibility and have the potential to be used in the bioprinting of neural tissue. The nano-chitosan/Ppy–alginate scaffold can be used as an ideal material for neural tissue engineering [71]. In the future, BP can combine with conductive polymers such as PPy for the repair of neural tissues and drug testing or precision medicine applications. Pan et al. [72] prepared injectable BPNS/CS/platelet-rich plasma (PRP) thermosensitive hydrogels. These hydrogels could control the release of BPNS degradation products, not only providing an ample supply of materials for bone regeneration but also clearing proliferative synovial tissue through PDT and PTT (Figure 3C). Furthermore, PRP enhanced the adhesion capacity of mesenchymal stem cells (MSCs). Experimental results clearly showed that this hydrogel significantly reduced joint swelling in arthritic mice, accelerated the bone repair process, and reduced friction on surrounding tissues to protect joint cartilage, offering a potential therapeutic approach for the treatment of arthritis.

#### 3.1.3. BP-Based Hydrogel Dressings

**Figure 4:**
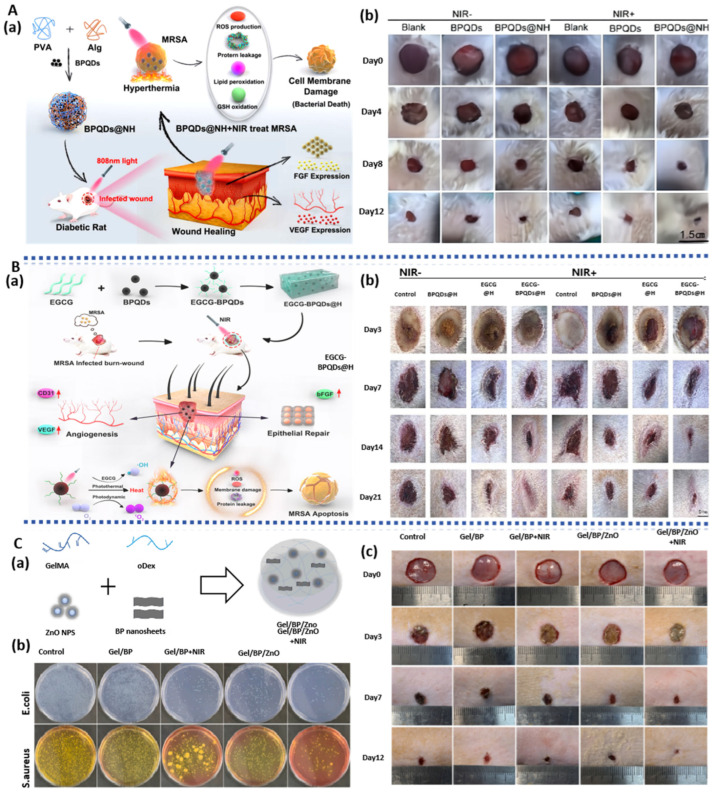
BP-based hydrogels for infected wound healing: (**A**): (**a**) Schematic illustration of the synthesis of BP-based hydrogels and antibacterial mechanism and their application in infected wound healing. (**b**) Photographs of MRSA-infected wounds in diabetic rats after different treatments at 0, 4, 8, and 12 days [75]. (**B**): (**a**) Schematic illustration of the synthesis of BP-based hydrogels and antibacterial mechanism and their application in infected wound healing. (**b**) Photographs of MRSA-infected wounds in diabetic rats after different treatments at 3, 7, 14, and 21 days [76]. (**C**): (**a**) Schematic illustration of the synthesis of BP-based hydrogels. (**b**) The effect of BP-based hydrogels with NIR irradiation on *S. aureus* and Ampr *E. coli*. (**c**) Photographs of bacterial-infected wounds in rats after different treatments at 0, 3, 7, and 12 days [73].

Hydrogels, as novel wound dressing materials, exhibit a high capacity for retaining moisture and excellent biocompatibility, effectively promoting cell migration and re-epithelialization. When combined with BP for its photothermal antibacterial effect, BP-based hydrogel dressings possess unique advantages in enhancing wound healing. Zhou et al. [73] prepared a broad-spectrum antibacterial BPNS/ZnO/Gel hydrogel through chemical cross-linking, where Zn induced polarization of macrophages toward the M2 phenotype (Figure 4C). M2 macrophages can secrete anti-inflammatory and cytokine factors to alleviate inflammation and promote neovascularization [74]. In vivo experiments have confirmed that, under the combined action of BP’s photothermal antibacterial effect and immune factors, this hydrogel significantly shortens the inflammatory phase, accelerates wound healing, and promotes the rapid formation of new blood vessels, demonstrating the ability to facilitate complete skin regeneration.

The treatment of chronic non-healing diabetic wounds and multidrug-resistant bacterial infections poses significant challenges in the clinical field. Enhancing the antimicrobial capacity of wounds is an effective approach to repairing chronic wounds. Huang et al. [75] employed a chemical cross-linking method to prepare a hydrogel composed of black phosphorus quantum dots (BPQDs), polyvinyl alcohol (PVA), and sodium alginate (Alg) that exhibited photothermal antimicrobial effects (Figure 4A). This gel generated reactive oxygen species (ROS), leading to glutathione depletion, bacterial lipid peroxidation, and, subsequently, the production of malondialdehyde, causing bacterial membrane rupture and exerting an antibacterial effect. Additionally, it regulated the expression of VEGF and basic fibroblast growth factor (bFGF), promoting wound healing in diabetic rats, with a wound closure rate reaching 95% after 12 days of treatment. Similarly, BPQDs modified with epigallocatechin gallate (EGCG) (BPDQs/EGCG) hydrogel demonstrated significant antimicrobial effects [76] (Figure 4B). The photothermal effect of BPQDs facilitated EGCG release and generated ^1^O_2_, synergistically exerting an antimicrobial effect, with an 88.6% killing rate against methicillin-resistant *Staphylococcus aureus* (MRSA). Moreover, the hydrogel significantly upregulated CD31 and bFGF, promoting the proliferation of vascular endothelial cells and epidermal cells. The wound healing rate in rats treated for 21 days was 92.4%, far surpassing the 61.1% in the control group. Furthermore, this hydrogel activated the PI3K/AKT and ERK1/2 signaling pathways to accelerate wound healing, providing theoretical support for the treatment of subsequent skin injuries. Mao et al. [77] immersed CS hydrogel in BPNS solution and prepared BPNS/CS hydrogel by freeze-drying. This hydrogel also activated the PI3K/AKT and ERK1/2 signaling pathways, promoting the healing of infectious wounds. BPNS rapidly and repeatably generated ^1^O_2_ under NIR irradiation, enhancing its antimicrobial activity against both Gram-positive and Gram-negative bacteria. In vivo experiments confirmed that this hydrogel accelerated wound closure significantly by promoting fibrinogen generation.

In addition to essential antibacterial treatments, the removal of ROS to enhance the wound’s antioxidative stress capability is also crucial for tissue damage repair. Ding et al. [78] initially prepared 4-octyl itaconate (4OI)-modified BPNS and further synthesized a gelatin composite hydrogel material (BPNS/4OI/Gel). Thanks to the PTT and PDT functions of BPNS, the BPNS/4OI/Gel hydrogel not only exhibits excellent antibacterial effects (with inhibition rates of 92.27% and 96.69% against *E. coli* and *S. aureus*, respectively) but also reduces the ROS levels within HUVECs cells from 62% to 29.8%, thereby enhancing antioxidative stress capabilities. BPNS/4OI/Gel hydrogel significantly improves the healing rate of full-thickness skin wounds in diabetic rats, with a wound closure rate of 99.64% on the 14th day (compared with 73.01% in the control group), demonstrating broad prospects in promoting skin wound healing.

#### 3.1.4. BP-Based Spray Gel

**Figure 5:**
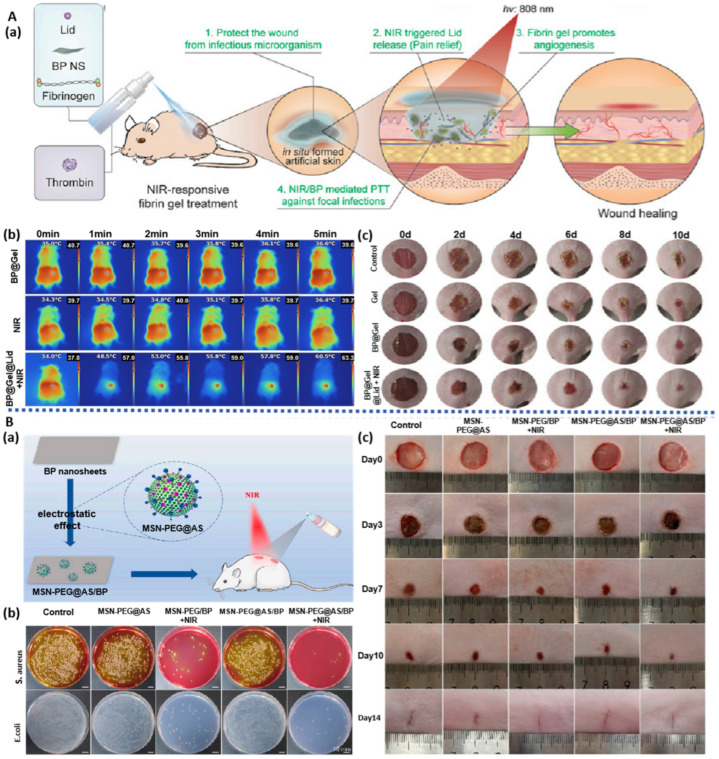
BP-based spray for infected wound healing: (**A**): (**a**) Schematic illustration of the construction of BP-based spray for bacteria-infected wound. (**b**) Changes in wound temperature within 5 min of exposure to near-infrared light. (**c**) Photographs of bacterial-infected wounds in diabetic rats after different treatments [81]. (**B**): (**a**) Schematic illustration of the construction of BP-based spray for bacteria-infected wounds. (**b**) Digital images of *E. coli* and *S. aureus* in vivo on the 3rd day after surgery. (**c**) Photographs of bacterial-infected wounds in diabetic rats after different treatments [82].

Spray-type hydrogels can adhere well to irregular wound surfaces and rapidly form a protective layer, preventing bacterial invasion. They offer the advantages of simplicity of application, controllable coating thickness, and rapid in situ gelation [79], making them highly suitable for on-site emergency wound treatment [80]. BP-based spray gels combine the characteristics of spray gels and BP, demonstrating significant advantages in reducing inflammation, providing sustained antibacterial effects, and accelerating wound repair. OuYang et al. [81] prepared a gel spray containing BPNS and lidocaine hydrochloride (Figure 5A). For one, the mixture of fibrinogen and thrombin in a dual-chamber spray device can rapidly form gelatinous fibrin at the wound site, achieving quick hemostasis. For another, this spray achieved a 94.3% clearance rate against *S. aureus* and promoted HUVEC proliferation and vascularization, which are favorable for wound healing. Moreover, BP-based spray gels possess NIR-responsive properties for controlled release of lidocaine hydrochloride, extending analgesia to 150 min, offering new possibilities for treating diabetic ulcers. Liu et al. [82] prepared a spray containing mesoporous silica nanoparticles (MSN) loaded with astragaloside IV (AS) through electrostatic adsorption and BPNS composites (Figure 5B). The photothermal effect of BPNS generates local high temperatures, causing covalent bond cleavage in polyethylene glycol (PEG) and releasing AS. This not only promotes macrophage polarization towards the M2 anti-inflammatory phenotype but also induces fibroblast proliferation, angiogenesis, and inflammation reduction. This spray achieved a sterilization rate of 99.58% against *E. coli* and 99.13% against *S. aureus*, demonstrating its antibacterial and anti-inflammatory properties and its ability to promote wound healing.

**Table 1:** A summary of the properties, functionalized modifications, and potential applications of BP-based hydrogel materials.

Materials	Modification	Property	Therapy Mode	Application	Ref.
BPNS	BP/PEA/GelMA hydrogel	Sustained supply of calcium-free phosphorus; Accelerate in situ mineralization deposition of osteocytes	hDPSCs; Rabbit model of cranial defects	bone regeneration	[44]
BPNS	BP/MgO/PVA/CS hydrogel	NIR photothermal antibacterial; Promote cell migration and osteogenic differentiation;	BMSCs; Rat model of cranial defects	bone regeneration	[63]
BPNS	BP/Gel hydrogel	NIR photothermal antibacterial; Eliminate the osteosarcoma cells; Enhance the bone regeneration capacity	hMSCs; Saos-2; Rat model of cranial defects	osteosarcoma; bone regeneration	[64]
BPNS	BP/DFO/Gel hydrogel	Superior swelling, degradation, and release rate; Promote neovascularization; Promote bone regeneration by activating the BMP/Runx 2 pathway;	BMSCs; Rat model of ischemic tibial defects;	ischemic bone defect	[65]
BPNS	BP/double network hydrogel	Promote cell proliferation and adhesion; Mechanical properties adjustable	hBMSCs; Rat model of cranial defects	bone regeneration	[66]
BPNS	BP/Mg double network hydrogel	Enhance early vascularization and neurogenesis; Promote bone regeneration and remodeling	HUVECs; NSCs; BMSCs; Rat model of bilateral skull defects	bone regeneration	[67]
BPNS	BP/CNT/OPF hydrogel	Conductive properties and syringeability; Enhance bone regeneration; Promote pro-osteoblast proliferation and differentiation	MC3T3-E1 cells; Rabbit model of Femur and spine defects	bone regeneration	[69]
BPNS	BP/CS/PRP hydrogel	Promote cell proliferation and adhesion; Protect the articular cartilage by reducing the friction; Generate ROS to suppress inflammation; Thermo responsive;	MSCs; Rat Model of rheumatoid arthritis.	rheumatoid arthritis	[72]
BPNS	BP/ZnO Gel hydrogel	Upregulate the expression of CD31 and α-SMA; Reduce inflammation and facilitate neovascularization	HUVECs; Rat model of full-thickness wound defect with bacterial infection	bacterial wound infections	[73]
BPQDs	BP/PVA/Alg hydrogel	NIR photothermal effect; ROS-generating and antibacterial; Reduce inflammatory response; Regulate the expression of VEGF and bFGF.	HUVECs; Rat model of diabetic wound infection	diabetic wound infections	[75]
BPQDs	BP/EGCG hydrogel	NIR photothermal effect; Generate ROS to suppress inflammation; Upregulate the expression of CD31 and bFGF; Promote wound healing by triggering the PI3K/AKT and ERK1/2 signaling pathways; Enhance cell proliferation and differentiation;	HUVECs; Rat model of diabetic burn-wound infection	diabetic wound infections	[76]
BPNS	BP/CS hydrogel	Generate ^1^O_2_ to suppress inflammation; Enhance the formation of the fibrinogen for accelerated incrustation; Trigger PI3K/Akt and ERK1/2 signaling pathways;	NIH-3T3 cells; Rat model of full-thickness wound defect with bacterial infection	bacterial wound infections	[77]
BPNS	BP/4OI/Gel hydrogel	Antibacterial and antioxidant; Promote neovascularization	HUVECs; Rat model of diabetic wound infection	diabetic wound infections	[78]
BPNS	BP/Lid/Gel spray	NIR photothermal antibacterial; Promote the proliferation of endothelial cells; Promote neovascularization; Relieve pain by NIR-triggered Lid release	HUVECs; Rat model of diabetic wound infection	diabetic ulcer	[81]
BPNS	BP/AS/MSN-PEG spray	NIR photothermal antibacterial; Promote neovascularization; Reduce inflammation	HUVECs; Rat model of full-thickness wound defect with bacterial infection	bacterial wound infections	[82]

### 3.2. BP-Based Scaffolds

Bone tissue engineering scaffolds have a series of advantages, including ease of shaping, convenient supply, and excellent mechanical properties. They have shown significant effectiveness in providing load-bearing capacity and interacting with the local extracellular matrix to promote bone healing. Their porous structure can mimic the layered pores of natural bone, facilitating the infiltration of the extracellular matrix into the scaffold, thereby enhancing bone healing capabilities. The combination of BP materials with osteoinductivity, photothermal efficiency, and antibacterial efficacy with synthetic polymers has driven the development of BP-based scaffolds. Currently, commonly used synthetic polymers in combination with BP materials in tissue engineering are mainly aliphatic polyesters such as polycaprolactone (PCL), polylactic acid–glycolic acid copolymer (PLGA), and polylactic acid (PLA). These polymers exhibit promising biocompatibility and biodegradability, making progress in promoting bone tissue regeneration and treating infectious bone defects and peripheral nerve injuries. Depending on the modification method of introducing BP into scaffold materials, they can be classified into BP surface-modified scaffolds and BP bulk-doped scaffolds (Table 2).

#### 3.2.1. BP Surface-Modified Scaffolds

BP surface-modified scaffolds are constructed by means such as electrostatic adsorption. Irregular and diverse forms of bone defects, along with the morphological inconsistency between implants and defect sites, can easily lead to surgical failure. Three-dimensional printing technology allows for the precise customization of scaffolds that match the patient’s defect site [83]. This technology enhances the mechanical strength of implants and the success rate of surgeries, representing an effective approach for fabricating biomimetic bone repair scaffolds [84]. Liu et al. [85] immersed amino-functionalized poly(propylene fumarate) (PPF) 3D-printed scaffolds in solutions containing graphene oxide (GO) and BPNS, followed by drying to prepare BPNS/GO/PPF scaffolds. The interconnected porous structure of this scaffold provides a microenvironment similar to the extracellular matrix. The introduced GO on its surface has a larger surface area and strong protein adsorption capability, promoting the adhesion and proliferation of MC3T3 cells on its surface and within its pores. Additionally, the degradation of BPNS, with the release of phosphate ions, significantly enhances bone regeneration and stimulates a notable increase in alkaline phosphatase (ALP) and osteocalcin (OCN) activities in MC3T3 cells. This approach holds the potential to expedite patient recovery.

**Figure 6:**
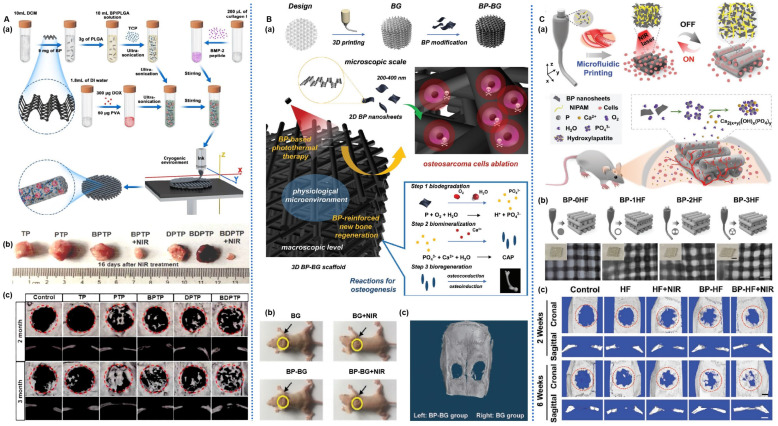
BP-based scaffold for bone regeneration: (**A**): (**a**) Schematic illustration of the fabrication process for BP-based scaffold. (**b**) Photographs of osteosarcoma after different treatments on day 16. (**c**) Micro-CT images of cranial defects implanted with different scaffolds for 2 and 3 months [109]. (**B**): (**a**) Schematic illustration of the fabrication process for BP-based scaffold and the stepwise therapeutic strategy for the elimination of osteosarcoma. (**b**) Photographs of osteosarcoma-bearing mice after different treatments on day 14. (**c**) Micro-CT images of harvested craniums obtained after treatments for 8 weeks [87]. (**C**): (**a**) Schematic illustration of the fabrication process for BP-based scaffold. (**b**) Schematic illustration and optical images. (**c**) Micro-CT reconstructed images of the harvested craniums obtained after treatments for 2 and 6 weeks [108].

In their study, Wu et al. [86] immersed hydroxyapatite (HA) scaffolds in a PLGA solution containing ZnL_2_/BPNS and evaporated the solvent to prepare BPNS/ZnL_2_/HA/PLGA scaffolds. The coordination between ZnL_2_ and BPNS not only prevented the oxidation of the P element, enhancing the stability of BPNS in the air, but also exhibited a synergistic photothermal sensitization and antibacterial effect. Consequently, this scaffold achieved effective antibacterial activity at temperatures below 50 °C (with a sterilization rate greater than 99% against *S. aureus* and *E. coli*), thus avoiding tissue damage from high temperatures. After infection elimination, the scaffold exhibited photo-thermal responsiveness and released Zn^2+^ and PO_4_^3−^, promoting the proliferation and osteogenic differentiation of human-bone-marrow-derived mesenchymal stem cells (hBMSCs) and accelerating new bone formation at the site of tibial defects. As bone tissue is one of the most common sites of tumor metastasis, BP composite scaffolds exhibit significant advantages by not only eliminating tumors but also promoting bone regeneration. Yang et al. [87] integrated BPNS into the 3D-printed bioactive glass (BPNS/BG) scaffold through surface modification (Figure 6B). In vivo experiments showed that this scaffold completely eliminated mouse osteosarcoma under NIR irradiation, with no tumor recurrence observed during the 14-day observation period. Subsequently, BPNS degradation produced phosphate, which drove calcium ion binding, accelerating biomineralization during bone formation. In contrast, mice implanted with conventional scaffolds experienced rapid tumor growth without new bone formation. Miao et al. [88] constructed a dynamic DNA hydrogel using VEGF-modified BPNS and infused it into 3D-printed PCL to create a BPNS/VEGF/DNA/PCL scaffold–hydrogel composite material. This material exhibited advantages such as customizability and high mechanical adaptability. Furthermore, it effectively harnessed photothermal release characteristics, with covalent bonds between BPNS and VEGF breaking to continuously release VEGF, accelerating angiogenesis. Both in vitro and in vivo experiments demonstrated that this composite material promoted MSC proliferation and differentiation, facilitating vascularization and osteogenic differentiation in cranial bone defects, ultimately improving the quality of engineered bone tissue.

In the utilization of BP materials, the thermally induced phase separation technique is employed to create high-molecular-weight scaffolds with a micro-porous structure and high surface area through the displacement of volatile solvents, thus enabling effective BP loading. Chen et al. [89] encapsulated BPNS loaded with strontium ions (Sr^2+^) and ibuprofen (IBU) into sodium alginate micro-spheres and incorporated them into a poly(ethylene imine)-modified PLLA (poly-L-lactic acid) nanofibrous scaffold prepared through thermally induced phase separation, resulting in the fabrication of a BP/IBU/SrCl2/PLLA scaffold. The photothermal effect of BP nanosheets imparts NIR-responsive release capabilities for Sr^2+^ and IBU to this scaffold, maintaining local drug concentrations to meet the practical requirements of bone repair processes. In vitro experiments have demonstrated cytocompatibility for this scaffold, with the sustained release of PO_4_^3−^ by BP significantly enhancing the scaffold’s in situ mineralization capacity, thereby promoting cell proliferation and bone tissue regeneration.

Zhao et al. [90] functionalized freeze-dried CS and PCL composite fiber webs (BPNS/PDA/Ag/CS/PCL) by integration of BPNS, modification of polydopamine (PDA), and in situ loading of silver (Ag) nanoparticles. Under NIR irradiation, the localized heating effect generated by BPNS promoted the expression of osteogenic-related proteins. Subsequently, the rapid, responsive release of Ag nanoparticles facilitated robust antibacterial capabilities both in vitro and in vivo, offering a novel approach for the treatment of infectious bone defects.

Compared with natural polymer scaffolds and synthetic scaffolds, metal scaffolds such as nickel (Ni), titanium (Ti), and magnesium (Mg) exhibit excellent mechanical properties and structural stability and have been widely used in the field of bone tissue engineering and repair. Ti and its alloys are known for their corrosion resistance and biocompatibility, often manufactured into porous structures extensively used for the production of bone tissue engineering scaffolds [91,92]. Ma et al. [93] prepared groove-like micro/nanostructures on the surfaces of Ni and Ti alloy scaffolds using femtosecond laser ablation. They further modified these scaffolds with biodegradable BPNS loaded with doxorubicin (DOX) through dip-coating and PDA chemical coating techniques. This modification improved the photothermal properties, biocompatibility, and chemical stability of the scaffold, enabling it not only to exhibit dual-controllable DOX release under NIR/pH conditions but also to possess potent antibacterial effects. It induced the death of tumor cells (Saos-2 and MDA-MB-231) in vitro, significantly accelerated the osteogenic process of mouse embryonic osteoblast precursor cells (MC3T3-E1), completely eliminated bone tumors in mice, and promoted significant bone regeneration. Xie et al. [94] deposited BPNS on Ti alloy surfaces through polydopamine oxidation self-polymerization, which significantly facilitated the repair of rat femoral defects. BPNS generated more ROS under dual stimulation of ultrasound and light, exerting an antibacterial effect (96.6% in vitro antibacterial rate against *S. aureus*, 97.3% in vivo antibacterial rate). This study revealed, for the first time, the significant impact of sono-dynamic therapy based on Ti/PDA/BPNS on bacterial membranes and oxidative stress at the transcriptome level. Yuan et al. [95] coated Ti scaffolds with a composite BPNS and HA layer for the treatment of bone infection (BPNS/HA/Ti). This coating exhibited a dual function of antibacterial activity and in situ biomineralization, promoting bone regeneration. BPNS had the ability to upregulate the expression of osteogenic markers such as ARS, ALP, OCN, and RUNX_2_. In vivo experiments also confirmed the effective repair of tibial defects by the scaffold and significantly suppressed the growth of *S. aureus* and *E. coli*, accelerating the healing process of infectious bone defects.

#### 3.2.2. BP Bulk-Doped Scaffolds

The concept of BP bulk-doped scaffolds involves the incorporation of BP into a precursor matrix of scaffolding material, achieved through a one-step process employing various techniques such as selective laser sintering, phase separation/cryogenic drying, microfluidic 3D printing, 4D printing, and layer-by-layer self-assembly. Selective laser sintering technology is one of the 3D-printing processes, where BP and its mixed powders are fused into thin layers through the thermal effect of high-energy laser beams, layer by layer, to form solid components. Wang [96] enhanced the structural stability of BPNS using a PDA-assisted oxidative self-polymerization method and subsequently introduced them into the PLLA scaffold matrix through selective laser sintering. With the introduction of BPNS/PDA, this scaffold not only meets the mechanical requirements for bone repair (with a 105% increase in compressive strength and a 50% increase in tensile strength) but also significantly improves the scaffold’s biocompatibility. This, in turn, promotes the adhesion, proliferation, and differentiation of human osteosarcoma cells (MG-63), showcasing significant potential applications in the field of bone tissue repair.

Phase separation/cryogenic freeze-drying technology involves freezing polymer solutions, emulsions, or hydrogels at low temperatures, inducing phase separation during the freezing process to create solvent-rich and polymer-rich phases. Subsequently, the solvent is removed through freeze-drying to form a porous structure. Depending on the system morphology, this technology can be categorized as emulsion freeze-drying, solution freeze-drying, or hydrogel freeze-drying. Li et al. [97] utilized a double emulsion method to prepare a solution of bone morphogenetic protein-2 (BMP-2)/PLGA microspheres, which were coated onto the BPNS/PLGA scaffold prepared using freeze-drying. This scaffold, through photothermal effects and the sustained release of BMP-2, upregulated the expression of HSP, thereby promoting osteogenic differentiation. The photothermal antimicrobial effect of BP endowed the scaffold with infection resistance. It exhibited promising biocompatibility and antibacterial properties both in vitro and in vivo, suggesting its potential for the treatment of infectious bone defects.

Qian et al. [98] subjected a BPNS/PCL dichloromethane solution to 20 min of ultrasonication and then sprayed it onto a rotating tubular mold through multiple nozzles of a spray gun. They employed a layer-by-layer self-assembly technique to fabricate well-biocompatible and highly conductive BPNS/PCL scaffolds, where pores were staggered between layers to facilitate interlayer cell adhesion and prevent the infiltration of foreign cells into the conduits, thereby ensuring normal axonal outgrowth, free exchange of water and oxygen. BPNS greatly improved tissue responses to electrical stimulation by enhancing the conduction of bioelectric signals, thus promoting the proliferation and differentiation of neural cells. Under the low ROS environment created by BPNS, this material induced angiogenesis and neurogenesis, stimulating calcium-dependent axonal and myelin sheath regeneration. After four months of in vivo implantation, peripheral nerve tissue regeneration extended up to 20 mm, offering a novel strategy for the treatment of long-distance nerve defects. Poly(3,4-ethylenedioxythiophene):polystyrene sulfonate (PEDOT:PSS) is a biocompatible conductive polymer comprising a positively charged PEDOT phase and a negatively charged PSS phase. The high conductivity of PEDOT:PSS, combined with its enhanced electrochemical stability, high biocompatibility, and easiness of processing when compared with other conductive polymers, has motivated its increasing use in several tissue engineering strategies. The polyacrylonitrile (PAN/PEDOT:PSS) nanofibers that were functionalized with apatite-like structure can simulate bone cell microenvironment and provide electrical stimulation, significantly promoting the proliferation of human bone oogenesis MG-63 cells and human bone marrow mesenchymal stem cells/stromal cells (hBM-MSCs), upregulating the expression level of bone marker genes during osteogenic differentiation of hBM-MSCs, highlighting its potential as an electroactive biomimetic BTE scaffold for innovative bone defect repair strategy [99]. The hBM-MSCs cultured on the PEDOT:PSS-coated polybenzimidazole (PBI) electrospinning scaffold for 1 week showed high viability, typical morphology, and proliferation ability [100]. In the future, BP can be combined with PEDOT: PSS materials to achieve the effect of promoting bone tissue repair.

Microfluidic technology offers advantages such as low cost, rapid processing, high throughput, and batch production, making it promising for the manufacture of high-quality bone scaffolds suitable for large-scale clinical applications [101,102,103,104,105]. Li et al. [106] employed microfluidic technology and 3D printing to construct a BPNS/HA-SiO_2_/PLLA scaffold using BPNS and HA-SiO_2_ microspheres. The stable pore connectivity of this scaffold facilitates the entry of BMSCs into the 3D fiber scaffold interior, promoting cell adhesion and proliferation. Furthermore, BPNS imparts the scaffold with photothermal responsiveness to enhance ion release, ensuring sustained delivery of Ca, P, and Si elements. This scaffold exhibits excellent performance in promoting rat cranial bone regeneration. Insufficient vascularization of newly formed bone tissue is a crucial factor hindering bone injury healing [107]. To enhance vascularization during bone reconstruction, Wang et al. [108] utilized microfluidic 3D printing to fabricate the BPNS-doped hollow fiber (BPNS/HF) scaffold (Figure 6C). With the incorporation of BPNS, this scaffold possesses reversible photothermal-responsive contraction and expansion properties (expansion below 40 °C and contraction above 40 °C), facilitating cell infiltration into the scaffold to promote blood vessel formation. Additionally, BPNS’s in situ biomineralization capability promotes osteogenic differentiation. In in vivo experiments, the channels formed by the BPNS scaffold facilitated deep infiltration of vessels into the scaffold, resulting in increased dense mature bone tissue, blood vessels, and central canals in the bone-deficient area, thereby accelerating the healing process.

In their study, Wang et al. [109] employed a low-temperature 3D printing approach to fabricate a stratified, porous, and mechanically robust scaffold using a water/PLGA/dichloromethane emulsion containing β-tricalcium phosphate (β-TCP), BPNS, DOX, and osteogenic peptides (Figure 6A). The synergistic effect of BPNS-mediated PTT and DOX resulted in the complete ablation of rat tumors with long-term recurrence inhibition. Subsequently, this scaffold facilitated the osteogenic differentiation of rat bone marrow mesenchymal stem cells (rBMSCs) and cranial bone regeneration, making it suitable for reconstructing bone defects post-bone tumor resection. Moreover, BPNS significantly mitigated the long-term toxicity associated with DOX, enhancing the clinical applicability and value of this scaffold.

Four-dimensional printing technology is also finding applications in bone tissue engineering. Its advantage lies in the fact that the scaffold can deform into the desired shape within a predetermined timeframe, combining the ease of implantation with minimally invasive procedures, thus achieving a more precise fit with irregular bone defect sites [110,111,112]. Wang et al. [113] incorporated BPNS and osteogenic peptides into β-tricalcium phosphate/poly(lactic acid–co-trimethylene carbonate) solution to prepare printing ink, producing the (BPNS/β-TCP/P(DLLA-TMC)) scaffold where shape and mechanical strength are both time-controlled. Due to the photothermal effect of BPNS, this scaffold reshapes at 45 °C, facilitating its implantation into irregular bone defects. After implantation, the scaffold rapidly decreases to 37 °C, exhibiting mechanical properties comparable to human cancellous bone, and slowly releases osteogenic active peptides, thereby enhancing osteogenic differentiation and bone regeneration capabilities at rat cranial defect sites.

**Table 2:** A summary of the properties, functionalized modifications, and potential applications of BP-based scaffold materials.

Materials	Modification	Property	Therapy Mode	Application	Ref.
BPNS	BP/GO/PPF 3D printed scaffold	Improve cell adhesion and proliferation; Release phosphate continuously; Stimulate cell osteogenesis	MC3T3 cells;	cell proliferation and osteogenesis stimulation	[85]
BPNS	BP/ZnL_2_/HA/PLGA 3D printed scaffold	NIR photothermal antibacterial; Photothermal osteogenesis; Prevent the recurrence of the bone tumors	hBMSCs; Rat model of tibial defects	Infectious bone defects	[86]
BPNS	BP/BG 3D printed scaffold	Ablate osteosarcoma by photothermal effect; Improve bone regeneration	hBMSCs; Bone tumor-bearing nude mice	osteosarcoma	[87]
BPNS	BP/VEGF/DNA 3D printed scaffold	Enhance the mechanical strength; Accelerate vascular regeneration and bone regeneration.	BMSCs; HUVECs; Rat model of cranial defects	vascularized bone regeneration.	[88]
BPNS	BP/IBU/SrCL_2_/PLLAscaffold	Promote cell adhesion and proliferation; Photothermal-responsive release drug Promote biomineralization in vitro; Promote cell proliferation	MC3T3-E1 cells;	bone repair	[89]
BPNS	BP/PDA/Ag/CS/PCL scaffold	Promote the expression of osteogenesis-related proteins; Excellent photothermal antibacterial;	rBMSCs; Rat model of femoral defects	Infectious bone defects	[90]
BPNS	BP/DOX/PDA/Fs-NiTi scaffold	Sufficient mechanical strength; Controllable drug release behavior of NIR/pH-dual sensitivity; Photothermal chemotherapy and photothermal antibacterial; Promote bone regeneration	tumor cells (Saos-2 and MDA-MB-231); Rat model of ectopic osteosarcoma	Osteosarcoma; Infectious bone defects	[93]
BPNS	BP/PDA/Ti scaffold	Photothermal antibacterial; Promote bone regeneration	BMSCs; Rat model of tibial defects	Infectious bone defects	[94]
BPNS	BP/HA/Ti scaffold	Photothermal antibacterial; Promote bone regeneration	BMSCs; Rat model of tibial defects	Infectious bone defects	[95]
BPNS	BP/PDA/PLLA scaffold	Improve the stability of the BPNS; Improve cell adhesion and proliferation; promote osteogenic differentiation.	MG-63 cells	Infectious bone defects	[96]
BPNS	BP/BMP-2/PLGA scaffold	NIR photothermal antibacterial; Photothermal osteogenesis;	PDSCs; Rat model of cranial defects	Infectious bone defects	[97]
BPNS	BP/PCL scaffold	Excellent biocompatibility and high conductivity; Induction of angiogenesis and neurogenesis	Schwann cells; Rat model of neurological defect	peripheral nerve injury	[98]
BPNS	BP/HA/SiO_2_/PLLA 3D printed scaffold	Photothermal effect promotes the release of elements; Accelerate osteogenesis.	BMSCs; Rat model of cranial defects	bone repair;	[106]
BPNS	BP/HF 3D printed scaffold	Promote osteogenic stem cell proliferation, differentiation, and mineralization; Enhance vascularized bone regeneration; NIR-response repeatable shrinkage/swelling performance;	rBMSCs; Rat model of cranial defects	bone defects; tissue engineering repairs	[108]
BPNS	BP/β-TCP/DOX/BMP2 3D printed scaffold	Sufficient mechanical strength; Excellent photothermal effect; Control drug release; Reduce the long-term toxicity phenomenon of released DOX in vivo; Promote osteogenesis	rBMSCs; Bone tumor-bearing nude mice	tumor resection-induced tissue defects.	[109]
BPNS	BP/β-TCP/P/P(DLLA-TMC) 4D printed scaffold	Photothermal-responsive shape memory; Suitable mechanical properties; Promote bone regeneration by the continuous release of peptides	rBMSCs; Rat model of cranial defects	bone defects of irregular shapes.	[113]
BPNS	BP/PEEK/PTFE scaffold	Excellent antibacterial properties and wear resistance.	*S. aureus*	Infectious bone defects	[114]

### 3.3. BP-Based Electrospun Fiber Membranes

Fiber membrane materials constructed through electrospinning technology possess a porous microstructure resembling the natural extracellular matrix. They also offer flexible and adjustable mechanical strength and a high surface area [115], which are conducive to cell migration, proliferation, adhesion, and differentiation [116,117]. Combining the excellent antibacterial, osteoinductive, and electrical signal conduction properties of BP, BP composite fiber membranes developed using this technology allow cells to migrate internally along interconnected pores. This not only compensates for the limitations of common materials in terms of cell penetration [118] but also provides mechanical support for subsequent blood vessel growth [119]. These properties make them highly suitable as implant materials for applications in the fields of neural, bone, and skin tissue engineering (Table 3).

**Figure 7:**
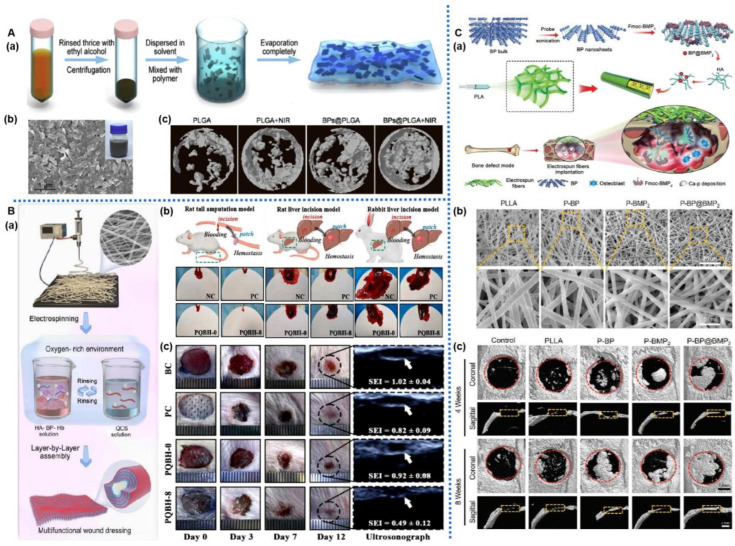
BP-based nanofiber for bone regeneration: (**A**): (**a**) Schematic illustration of the construction of BP-based membrane for bone regeneration. (**b**) SEM image of BP-based membrane. (**c**) Micro-CT images of the tibia defect after different treatments for 5 weeks [130]. (**B**): (**a**) Schematic illustration of the construction of BP-based nanofiber for infected wound healing. (**b**) Images of BP-based nanofibers hemostatic condition in three models. (**c**) Photographs of bacterial-infected wounds in diabetic rats after different treatments at 0, 3, 7, and 12 days [129]. (**C**): (**a**) Schematic illustration of the construction of BP-based nanofiber for bone regeneration. (**b**) SEM image of PLLA and BP-based nanofiber. (**c**) Micro-CT images of the cranial defect after different treatments for 4 and 8 weeks [121].

Lee et al. [120] employed electrospinning technology to fabricate BPQDs/PCL/collagen (Col) nanofiber membranes. These membranes exhibited excellent biocompatibility, facilitating the proliferation and adhesion of MC3T3-E1 cells and promoting cell osteogenic differentiation by upregulating OCN and ALP levels. Loading BMP-2, VEGF, and other active factors onto BPNS expedited the process of bone regeneration. Cheng et al. [121] utilized a sol–gel electrospinning technique to incorporate BMP-2-modified BPNS onto PLLA electrospun fiber scaffolds (Figure 7C). BMP-2 selectively recruited and induced osteogenic differentiation of osteoprogenitor cells, while BPNS accelerated biomineralization, achieving staged bone regeneration. When implanted in a rat cranial defect model, these membranes demonstrated excellent biocompatibility and strong osteogenic capabilities. After 8 weeks of treatment, nearly complete new bone formation was observed at the site of the rat bone defect. Wang et al. [122] used a similar strategy to prepare biocompatible BPNS-VEGF-PLLA nanofiber membranes, which promoted BMSCs osteogenic differentiation and vascular endothelial cell angiogenesis and accelerated the bone defect repair process.

Sensory nerves are abundantly present in the periosteum, cortical bone, and bone marrow cavity, with nerve axons gradually extending into the bone marrow through nutrient foramina [123]. Sensory neurons act directly on hematopoietic stem cells (HSCs) by secreting neuropeptides, such as calcitonin gene-related peptide, receptor activity-modified protein1, and calcitonin receptor-like receptors via the activation of Gαs/adenylate cyclase/cAMP pathways that stimulate downstream HSCs [124]. Several neuropeptides, which are secreted by sensory nerves, facilitate bone reconstruction [125]. Therefore, Su et al. [126] utilized coaxial electrospinning technology to fabricate (core)–polycaprolactone (PCL)/s(shell)–DNM biomimetic periosteum(PD). BPNS combined with PD through electrostatic interactions, constructing an electrically active biomimetic bone membrane (BPNS/PD). The conductivity of BPNS provides electrical stimulation for sensory nerve regeneration, while the decellularized neurotransmitter offers an appropriate extracellular matrix for nerve regeneration. This membrane stimulates Schwann cells through the Von Willebrand factor pathway to transform into a reparative phenotype, enhances the excitability of sensory neuron axon initial segments, regulates dense core vesicle transport, and promotes neurotransmitter release. These effects facilitate the growth of neuronal axons in the bone marrow, vesicle secretion, and the osteogenic transformation of BMSCs, significantly promoting cranial bone growth in mice and offering a novel approach for the treatment of bone defects.

In the field of bone tissue engineering, the photothermal effect of BPNS demonstrates excellent therapeutic efficacy, offering advantages such as reduced infection rates and promotion of bone regeneration. Zhang et al. [127] utilized electrospinning techniques to prepare BPNS/PCL nanofiber scaffolds, which were further surface-functionalized with aptamers (Apt19s) after oxygen plasma treatment. Subsequently, phase-change material particles loaded with vancomycin were deposited onto the scaffold surface to create the Apt-PCL/BP scaffold. Under NIR irradiation, the photothermal effect of BPNS triggered a solid-liquid phase transition, thereby releasing vancomycin, resulting in significant antibacterial effects both in vitro and in vivo. Apt19s recruited MSCs at the injury site, and the photothermal effect of BPNS upregulated HSP while continuously releasing P element, which could promote MSCs’ osteogenic differentiation and biomineralization and expedite the repair process of cranial bone injuries in rats.

The antibacterial properties of BP exhibit unique advantages in the field of skin tissue engineering, with the ability to promote wound healing by preventing bacterial infections. Inspired by existing naturally superhydrophobic surface structures, Zhao et al. [128] utilized electrospinning techniques to fabricate an asymmetric wettable bilayer membrane containing BP (BPNS/PLGA/ginsenoside Rg1 (Rg1)/Gel fiber membrane). This fiber membrane consists of a hydrophobic outer layer (PLGA and BP graft chitosan) and a hydrophilic inner layer (Gel and Rg1), demonstrating excellent mechanical properties and suitable moisture content. This not only promotes cell proliferation and water vapor permeation but also exhibits remarkable antibacterial effects. Due to the excellent antibacterial activity of BP, the hydrophobic outer layer prevents bacterial colonization, while Rg1 in the hydrophilic inner layer promotes the migration of HUVECs and vascular formation. By triggering the phosphoinositide 3-kinase (P-PI3K/PI3K) and protein kinase B (P-AKT/AKT) signaling pathways, this fiber membrane upregulates the protein expression of Ki67, CD31, α-SMA, and TGF-β1; inhibits inflammation; and promotes collagen deposition, cell proliferation, and blood vessel formation, ultimately facilitating wound healing. Severe hypoxic microenvironments, often caused by high glucose levels, local bleeding, and bacterial infections, can lead to the formation of hyperproliferative scars. To address this, Zhao et al. [129] used electrospinning techniques to construct a PLLA/quaternized chitosan (QCS)-based fiber membrane, which was further assembled layer by layer with positively charged quaternized chitosan solution and negatively charged hyaluronic acid/BPNS/hemoglobin solution (Figure 7B). BPNS’s photothermal effect stimulated the in situ release of oxygen from oxygen-carrying hemoglobin (Hb), improving oxygen deficiency at the injury site and promoting cell proliferation, migration, and vascularization. The hemostatic and broad-spectrum antibacterial properties of quaternized chitosan further expedited the wound healing process, enhancing wound closure rates.

**Table 3:** A summary of the properties, functionalized modifications, and potential applications of BP-based nanofiber materials.

Materials	Modification	Property	Therapy Mode	Application	Ref.
BPQDs	BP/PCL/Col nanofiber	Promote cell attachment and proliferation; Improve osteogenic differentiation	MC3T3-E1 cells	osteodifferentiation enhancement	[120]
BPNS	BP/BMP2/PLLA nanofiber	Staged bone regeneration; Accelerate biomineralization;	BMSCs; Rat model of cranial defects	bone repair	[121]
BPNS	BP/VEGF/PLLA nanofiber	Promote osteogenic differentiation and angiogenesis	rBMSCs HUVECs	bone repair	[122]
BPNS	BP/PD nanofiber	Induce neurogenesis; Promote osteogenic differentiation; Stimulate Fanconi anemia pathway,	BMSCs; Schwann cells; Rat model of cranial defects	bone regeneration; neurogenesis	[126]
BPNS	BP/Apt19s-PCL nanofiber	Antibacterial through photothermal-triggered drug delivery; Accelerate biomineralization	MSCs Rat model of cranial defects	bone repair	[127]
BPNS	BP/Rg1/PLGA/Gel nanofiber	NIR photothermal antibacterial Facilitate the migration and tube formation of HUVECs Promote M2 polarization of macrophages; inhibit M1 polarization of macrophages.	3T3 cells; HUVECs; Rat model of full-thickness wound defect with bacterial infection	wound healing	[128]
BPNS	BP/Hb/PLLA nanofiber	NIR-assisted oxygen delivery; Hemostasis; NIR photothermal antibacterial; Reduce inflammation; Promote cell proliferation, migration, and vascularization.	HUVECs Mouse fibroblasts (L929) Rat tail amputation Rat liver injury Rabbit liver injury models	wound healing	[129]
BPNS	BP/PLGA membrane.	Heat-induced osteogenesis; NIR photothermal response	Rat model of tibia defect	bone tissue engineering	[130]

### 3.4. BP-Based Microspheres

Drug-loaded microspheres can physically encapsulate or adsorb drugs on their surface, and their advantages, such as targeted delivery, controlled release, and low irritability, contribute to maintaining therapeutic drug concentrations at the site of disease [131], reducing the dosing frequency and improving patient compliance with medication. The porous structure of microspheres facilitates cell adhesion and proliferation, making them widely applicable as cell carriers in irregular tissue defect repair. Based on their structure, microspheres can be classified into porous microspheres, bilayer microspheres, and magnetic microspheres, which can be prepared through methods such as emulsification and evaporation, phase separation, spray drying, and hot-melt extrusion [132,133]. By encapsulating BPNS with antibacterial and photothermal effects within microspheres, not only can the material’s mechanical strength and porosity be enhanced, but the drug release behavior can also be modulated, bacterial growth can be inhibited, and tissue regeneration can be regulated. BP-based microspheres hold great potential as tissue engineering materials (Table 4).

**Figure 8:**
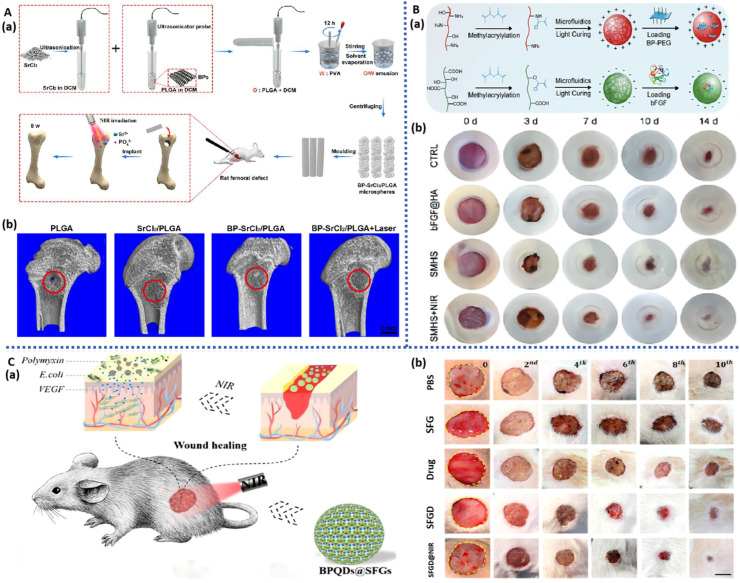
BP-based microparticles for tissue engineering: (**A**): (**a**) Schematic illustration of the construction of BP-based microparticles for bone regeneration. (**b**) Micro-CT images of the femoral defect after different treatments for 8 weeks [134]. (**B**): (**a**) Schematic illustration of the construction of BP-based microparticles for wound healing. (**b**) Representative images of the wound healing process in diabetic rats [136]. (**C**): (**a**) Schematic illustration of the construction of BP-based microparticles for infected wound healing. (**b**) Photographs of bacterial-infected wounds in diabetic rats after different treatments [135].

The photothermal effect of BP imparts microspheres with photothermal responsiveness, conferring a distinct advantage in precisely controlling drug release. Given the importance of Sr elements in promoting bone defect repair, Wang et al. [134] prepared photothermal-responsive BPNS/SrCl_2_/PLGA microspheres using a water-in-oil emulsion solvent evaporation method, which demonstrated excellent tissue compatibility upon implantation at the site of rat femoral defects (Figure 8A). Under NIR irradiation, the outer PLGA shell underwent thermal decomposition, thereby precisely releasing Sr^2+^, further stimulating osteogenic cell differentiation and bone formation, and manifesting exceptional bone regeneration capability.

To address the limitations of local administration of VEGF and antimicrobial peptides, Zhang et al. [135] encapsulated BPQDs, VEGF, and antimicrobial peptides together within gelatin and silk fibroin microspheres for controlled drug release and wound healing (Figure 8C). The photothermal effect of BPQDs resulted in the melting of gelatin, imparting NIR-responsive release characteristics for VEGF and antimicrobial peptides. Consequently, these microspheres demonstrated the ability to promote neovascularization and exhibit antimicrobial properties, thereby displaying a commendable capacity to enhance wound healing in a rat skin infection model.

BP-based hydrogel microspheres, characterized by their small and controllable size, uniform spherical structure, and straightforward fabrication process, have significant potential in the treatment of diabetic wounds and other tissue regeneration applications. Luo et al. [136] developed hydrogel microspheres (BPNS/CS/bFGF/HA) through charge interaction by loading positively charged CS microspheres with BPNS and negatively charged hyaluronic acid (HA) microspheres with bFGF (Figure 8B). The photothermal responsiveness of BPNS regulates the degradation rate and drug release location of the microspheres, showing a concentration-dependent effect. In vitro experiments demonstrated excellent biocompatibility of these microspheres, promoting the proliferation of HUVECs and mouse embryonic cells (NIH/3T3), as well as macrophage polarization. In diabetic rat skin wounds, these microspheres, through PTT and chemical intervention, synergistically suppressed inflammation, facilitated vascularization, and promoted tissue remodeling, significantly accelerating the regeneration of diabetic wounds.

### 3.5. BP-Based Microneedles

Microneedles, consisting of multiple micrometer-sized needle tips arranged in an array on a base, offer a distinct advantage over conventional transdermal patches and creams. Microneedles can penetrate the stratum corneum in a targeted manner without engaging the pain receptors, creating microscale mechanical channels through which drugs are released into the epidermis or dermis. This involvement in the microcirculation induces pharmacological responses, significantly enhancing the transdermal rate and absorption of drugs, especially large molecules [137,138]. Henry et al. were the first to apply microneedles in the mainstream drug delivery field, sparking a boom in the industrialization of microneedles [139]. The length, height, and shape of microneedles can be individually tailored according to therapeutic requirements [140,141], and they can be categorized based on their morphology as solid, hollow, coated, dissolvable, and hydrogel microneedles. Microneedles based on BP, which exhibit photothermal conversion properties, have garnered significant attention in the field of skin tissue repair [142]. Currently, there are four main methods of transdermal drug delivery: pre-attachment followed by adhesion (solid microneedles), pre-penetration followed by release (dissolvable and freeze-dried microneedles), coating-based delivery (coated microneedles), and injection-based delivery (hollow and gel microneedles).

**Figure 9:**
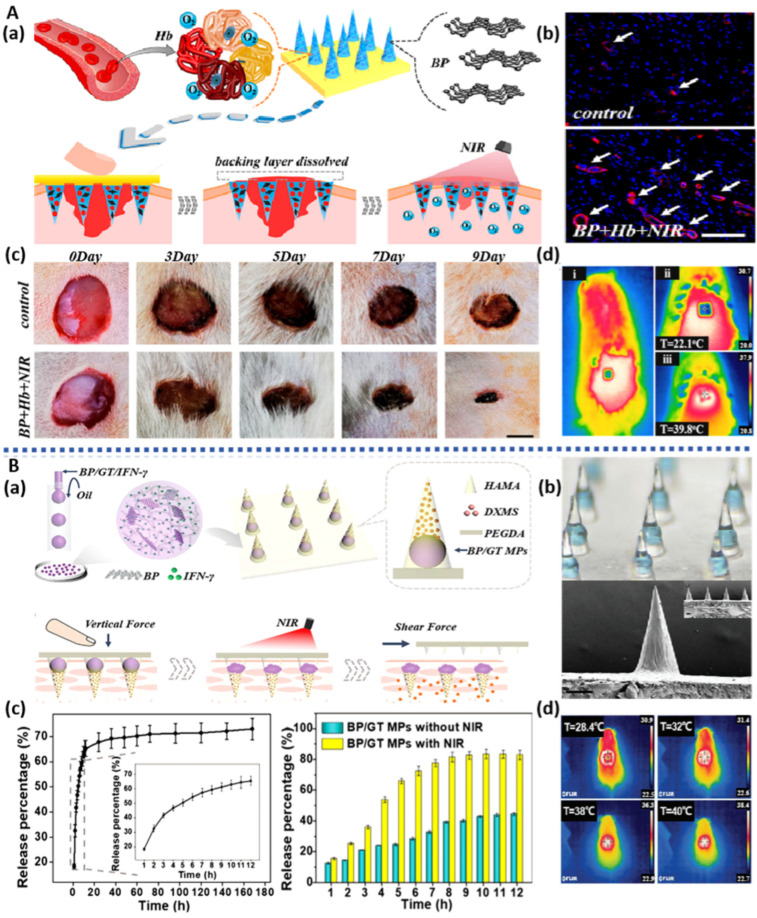
BP-based microneedles for infected wound healing: (**A**): (**a**) Schematic illustration of the construction of BP-based microneedles for infected wound healing. (**b**) Corresponding double immunofluorescent staining of CD31 and α-SMA on day 9. The arrows indicate the vascular ducts. (**c**) Photographs of bacterial-infected wounds in diabetic rats after different treatments. (**d**) Thermal images of BP-based microneedles applied to the rat dorsal skin before and after 2 min NIR irradiation [143]. (**B**): (**a**) Schematic illustration of the construction of BP-based microneedles for infected wound healing. (**b**) Optical microscopy images and SEM images of BP-based microneedles. (**c**) The accumulative Rhodamine B and FITC-BSA release from BP-based microneedles. (**d**) Thermal images of the mouse dorsal skin applied with the BP-based microneedles [144].

The photothermal effect of BP imparts controllable release characteristics to microneedles, offering convenience for the repair of skin defects. Zhang et al. [143] developed soluble microneedles with controllable oxygen delivery capability (BPQDs/Hb) for the treatment of wound injuries (Figure 9A). These microneedles were composed of a PVA material as the base, GelMA material at the tip, and loaded with BPQDs and Hb at the tip. The photothermal effect of BPQDs increased the temperature of the microneedles, causing the base to dissolve while the tip remained in the wound. Upon heating, Hb’s oxygen-binding capacity decreased, leading to controlled oxygen delivery, significantly improving the wound’s oxygen deficiency condition and demonstrating a remarkable therapeutic effect on full-thickness skin defects in diabetic rats. Fan et al. [144] used capillary microfluidic technology to prepare BPNS/Gel microspheres, which were loaded into hydrogel microneedles via UV curing (Figure 9B). Under the influence of BPNS photothermal effects, the microspheres turned into a liquid, creating a cavity inside the microneedle. This resulted in the separation of the base and the needle tip under shear forces, allowing for the continuous release of dexamethasone into the skin. This controllable separation of microspheres–microneedles enabled the loading and release of two types of drugs, expediting the healing process in mice with systemic lupus erythematosus (Table 4).

Blood supply is crucial for the formation of skin; therefore, in the field of skin tissue engineering, there is a growing emphasis on vascular repair. Inspired by the concealed spines and inflation-deflation properties of pufferfish, Zhang et al. [145] employed an immersion and rolling-assisted template replication method to construct three-layered micro-needle balloons containing BP and rapamycin (BP/rapamycin). The upper and lower layers consisted of gelatin containing BP, while the middle layer enveloped rapamycin-loaded micro-needles. Protected by gelatin, the BP/rapamycin micro-needle balloons effectively reached the lesion site. Under NIR irradiation, the photothermal effect of BP melted the gelatin shell, achieving the separation of the micro-needles from the balloon catheter. This ensured the sustained release of rapamycin after catheter removal, significantly improving the condition of aortic restenosis in rats. Small vascular lesions and dysfunction of fibroblast cells can easily lead to skin fibrosis. To alleviate this condition, Luan et al. [146] employed a template-assisted layer-by-layer curing method to prepare photothermal-responsive micro-needles. The needle tips encapsulated triptolide and paeoniflorin (TP/Pae), while the needle bases encapsulated BP/TP/Pae. BP’s excellent photothermal effect endowed the micro-needles with the ability to thermally release drugs. Combined administration of TP and Pae not only exhibited anti-inflammatory, detoxification, and immune-regulating effects to alleviate inflammation-induced skin fibrosis but also significantly reduced the toxicity of drug delivery. Experiments demonstrated that these micro-needles notably improved mouse skin fibrosis and capillary dilation and reduced collagen deposition and epidermal thickness, showcasing tremendous potential in the field of skin tissue engineering.

The uniform distribution of adipose tissue plays a crucial role in skin tissue engineering. Peng et al. [147] demonstrated the construction of a BP-modified rosiglitazone (Rosi)-loaded soluble microneedle patch (BPNS/Rosi) using laser etching for dynamic regulation of adipose tissue. Due to the excellent photothermal effect of BPNS, these microneedles rapidly and continuously heated under NIR irradiation, maintaining a temperature of 40–50 °C, thereby facilitating the efficient and painless delivery of Rosi into the target adipose tissue within the microneedles and assisting in the lysis of adipocytes. After one month of treatment, the body weight of high-fat diet-induced obese mice decreased by approximately 8%, and their waist circumference decreased by approximately 21%, demonstrating precise and efficient fat reduction effects.

### 3.6. BP-Based Liposomes and Vesicles

**Figure 10:**
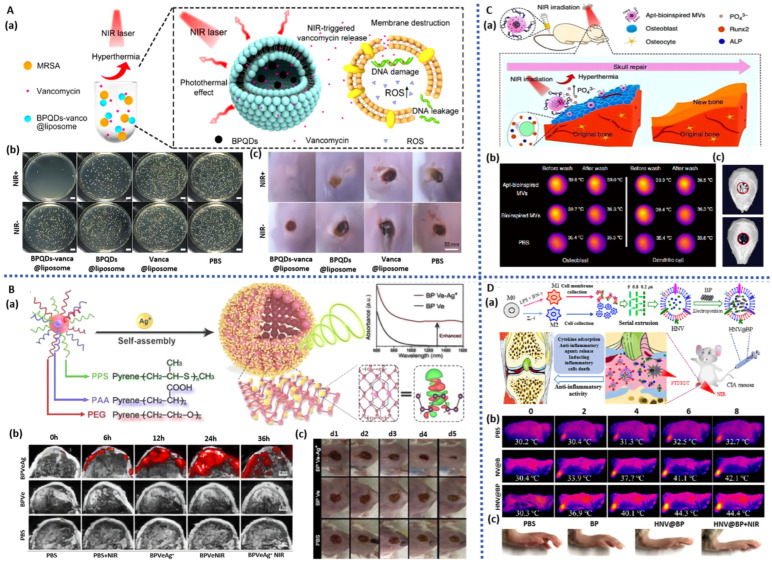
BP-based liposome and vesicles for infected wound healing and arthritis. (**A**): (**a**) Schematic illustration of the construction of BP-based liposome and its antibacterial mechanism for infected wound healing. (**b**) Antibacterial activity in vivo on the 3rd day after surgery. (**c**) Photographs of bacterial-infected wounds in diabetic rats after different treatments [151]. (**B**): (**a**) Schematic illustration of the construction of BP-based vesicles for wound healing. (**b**) The NIR-II photoacoustic images of tumor in mice. (**c**) Photographs of bacterial-infected wounds in diabetic rats after different treatments [152]. (**C**): (**a**) Schematic illustration of the construction of BP-based vesicles for bone regeneration. (**b**) Infrared images of rat osteoblasts and dendritic cells. (**c**) Micro-CT images of the cranial defect after different treatments [153]. (**D**): (**a**) Schematic illustration of the construction of BP-based vesicles for bone arthritis. (**b**) Photothermal images of arthritis sites upon NIR irradiation after 24 h post-injection of PBS, NV@BP, and HNV@BP. (**c**) Representative images of arthritic paws from different groups [154].

Liposomes and vesicles are of great interest due to their bilayer phospholipid encapsulation structure, offering high biocompatibility, biodegradability, and the ability to control the release of drugs through various administration routes, making them an ideal choice for drug delivery carriers. They can be loaded with both hydrophilic and hydrophobic molecules in their core and membrane, forming nanocarriers [148], which can enter the body through multiple administration routes, such as oral, injection, and localized transdermal delivery, significantly reducing the degradation of payloads in circulation and extending their circulation time [149,150]. They have been widely used in drug delivery systems and tissue engineering. However, liposomes and vesicles still face challenges, such as difficulties in granulation and slow drug release after transporting drugs to the target site, posing inconveniences in clinical applications. The local hyperthermia generated by BP disrupts the carrier structure, enabling the release of loaded drugs at the target site. Therefore, BP-based liposomes and vesicles, as photothermal-responsive drug delivery systems, hold enormous potential in applications such as antibacterial therapy, skin repair, and bone regeneration (Table 4). Zhang et al. [151] loaded BP quantum dots and vancomycin (BPQDs/vanco) onto thermosensitive liposomes prepared using a thin-film hydration method (Figure 10A). These liposomes, influenced by BPQDs’ photothermal effect, released vancomycin at the site of skin injuries, significantly improving antibacterial efficiency by disrupting MRSA cell membranes, causing protein leakage, elevating bacterial ROS levels, and damaging DNA. They demonstrated excellent curative effects against skin abscesses caused by antibiotic-resistant bacteria, providing insights into the mechanisms of skin injury repair.

In order to improve the defects of BPQDs’ low absorbance in the second infrared region and poor optical imaging ability, Li et al. constructed BPQDs/polyacrylic acid (PAA)/PEG/polyphenylene sulfide (PPS) vesicles by non-covalent modification for the first time. Ag^+^ was modified on the surface of the vesicles by hydrophobic interaction to improve the ability of biological imaging BPQDs and explore their potential in diversified treatment [152] (Figure 10B). Ag^+^ reduces the bandgap of BPQDs through strong charge coupling, thereby enhancing its absorption intensity in the second infrared region. The vesicles accurately release Ag^+^ in the lesion area, which not only kills pathogenic bacteria and accelerates wound healing but also can be captured by macrophages, causing a strong immune response of the body and then producing ROS to induce immunogenic cell death, thus serving a therapeutic role in cancer diagnosis and treatment.

Wang et al. [153] encapsulated BPQDs into PLGA nanospheres and modified them with nucleic acid Apt for specific recognition of osteoblasts, thereby constructing BPQDs/Apt/PLGA vesicles. Apt precisely targeted osteoblasts, where BPQDs degraded to generate PO_4_^3−^, promoting cellular biomineralization and upregulating the expression of HSP and ALP through photothermal effects, further enhancing the bone regeneration process (Figure 10C). Upon implanting the vesicles into a murine cranial defect model, substantial new bone formation occurred both in the periphery and at the center, leading to nearly complete healing of the bone defect. Zhao et al. [154] fused membranes of M1-type macrophages with those of M2-type macrophages to prepare nanovesicles (HNV) and subsequently introduced BPNS using an electroporation system for treating rheumatoid arthritis (Figure 10D). Due to HNV retaining the anti-inflammatory properties of M2-type macrophages and the cytokine receptors of M1 membranes, the photothermal effects of HNV and BPNS synergistically exerted anti-inflammatory effects, demonstrating a significant therapeutic effect on collagen-induced rheumatoid arthritis (RA) in mice.

**Table 4:** A summary of the properties, functionalized modifications, and potential applications of other BP-based materials.

Materials	Modification	Property	Therapy Mode	Application	Ref.
BPNS	BP/SrCl2/PLGA microspheres	NIR-triggered drug delivery system; Improve bone regeneration by photothermal effect	Rat model of femoral defects	bone repair	[134]
BPQDs	BP/silk fibroin/gelatin microspheres	Promote neovascularization; NIR photothermal antibacterial; NIR-triggered drug delivery system	HUVECs; Rat model of full-thickness wound defect with bacterial infection	drug delivery and wound healing.	[135]
BPNS	BP/CS/bFGF/HA microspheres	Promote neovascularization and wound healing; NIR-triggered drug delivery system	HUVECs NIH/3T3 cells Rat model of diabetic wound infection	wound healing	[136]
BPQDs	BP/Hb separable microneedles	NIR responsive oxygen delivery; Promote wound healing	NIH 3T3 cells; Rat model of diabetic wound infection	drug delivery; wound healing	[143]
BPNS	BP/GT separable microneedles	NIR-regulated separable microneedles; Promote wound healing	NIH 3T3 cells; Rat model of systemic lupus erythematosus	systemic lupus erythematosus; drug delivery and wound healing	[144]
BP	BP/rapamycin microneedle balloon catheters	NIR-triggered drug delivery system Improved abdominal aortic restenosis	Rat model of abdominal aorta restenosis	wound healing	[145]
BP	BP/TP/Pae separable microneedles	NIR photothermal antibacterial; NIR-triggered drug delivery system; Relieve the fibrosis of the skin	NIH-3T3 cells; Rat model of early full-thickness skin wound	wound healing; vascular fibrosis	[146]
BPNS	BPNS/ROsi separable microneedles	NIR-triggered drug delivery system; Regulating the uniform distribution of the adipose tissue	mouse fibroblasts (L929) and human fibroblasts (HSF); C57 mice model of induced by a high-fat diet	wound healing; reduce weight	[147]
BPQDs	BP/vanco liposome	NIR photothermal antibacterial; NIR-triggered drug delivery system;	*Staphylococcus aureus*; Rat model of bacteria-infected subcutaneous abscess	antibiotic-resistant bacteria-caused skin abscess	[151]
BPQDs	BP/Ag^+^ vesicles	NIR-II photoacoustic imaging capability; NIR photothermal antibacterial; Photodynamic therapy	dendritic cells (DCs); bilateral4T1-tumor-bearing BALB/c mice. Rat model of bacteria-infected	immunization therapy; wound healing	[152]
BPQDs	BP/Apt/PLGA vesicles	Guide molecular recognition; NIR photothermal effect; Promote biomineralization	In vitro cell experiment; Rat model of cranial defects	bone repair	[153]
BPNS	BP/HNV vesicles	NIR photothermal antibacteria; NIR-triggered drug delivery system	RAW264.7 cells; L929 cells; Rat model of collagen-induced arthritis	bone arthritis	[154]

## 4. Discussion

Black phosphorus (BP), as a novel two-dimensional biocompatible nanomaterial, not only exhibits excellent optoelectronic properties but also possesses remarkable biocompatibility and degradation capabilities, rendering it highly promising in biomedical fields such as tumor therapy and tissue regeneration. Furthermore, BP nanomaterials have a wide range of raw material sources, and large-scale production processes continue to improve, laying a solid foundation for their industrial applications. This article systematically reviews the research progress of BP-based composite medical materials in the field of tissue engineering. It elaborates on the material design and mechanism of action of BP composite materials in bone regeneration, skin repair, nerve regeneration, and inflammation treatment based on the physicochemical and biological characteristics of BP materials. As research on BP materials and BP composite biomedical materials deepens in terms of preparation methods, structural design, and biological effects, this field is showing more significant advantages in applications and a broader potential for industrialization.

## 5. Prospects

To expand the use of BP in medicine and other fields. In the future, further exploration can be pursued in the following areas:

(a) BP composite biomedical materials exhibit excellent photothermal antibacterial properties and drug-release behavior, providing significant therapeutic advantages for challenging chronic wounds. With the development of precision medicine and smart healthcare concepts, intelligent BP-based diagnosis and treatment platforms that integrate advanced biosensing technologies for real-time monitoring, remote healthcare, and on-demand drug delivery represent an important future direction in this field.
(b) BP readily oxidizes to form harmless phosphate compounds in the human body [155], possessing unique advantages in terms of biocompatibility. However, the long-term stability of BP-based composite biomedical materials is relatively poor during their preparation and storage processes. At present, the reported strategies to improve the stability of BP include physical encapsulation methods, such as coating with thin films of aluminum oxide, tin oxide, graphene, and hexagonal boron nitride [156], as well as surface chemical modification methods, such as polyethylene glycol [157], aromatic diazonium, metal ions, and metal–ligand modifications [158,159]. Nevertheless, most of these strategies face challenges related to high application costs, low protection efficiency, and complex operations. Developing economically efficient strategies to enhance the long-term stability of BP materials is a critical issue that urgently needs to be addressed for industrial applications. Innovative approaches, such as doping methods [160] or the application of biofilms [33], can be considered to make BP nanomaterials more practical for real-world use.
(c) BP composite biomedical materials have demonstrated vast potential in the field of tissue engineering, addressing the non-self-supporting defects of BP nanomaterials. In dealing with irregular motion-type traumas, the integration of 3D and 4D printing technologies, electrospinning techniques, and layer-by-layer self-assembly techniques offers the prospect of producing BP composite biomedical materials that are better suited for clinical application. Adaptable control over processing techniques and materials based on the type of trauma allows for continued exploration of novel materials on the foundation of existing hydrogels, scaffolds, and fiber membranes. However, further exploration is needed on how to optimize the controllable preparation of BP nanomaterials and the production costs of BP composite medical formulations, as well as scaling up the production process. For example, although microsphere formulations can bypass the first-pass effect, improve bioavailability, and reduce drug dosage [161], they present high technological barriers, high development costs, and long cycles. Aside from addressing issues related to encapsulation rates and particle size uniformity, ensuring the sterility of the production process is also critical. Research into BP-based microsphere materials is limited, but future investigations in the fields of BP magnetic microspheres and bilayer microspheres can be pursued to enhance their performance [162]. Questions regarding whether BP-based microneedles will cause destructive damage to the skin and whether the voids formed in the skin are reversible remain to be verified. These factors have constrained the clinical application of microneedle technology, necessitating the establishment of unified standards for microneedle technology to enhance safety [163].

Despite significant advancements in the research of BP composite biomaterials for tissue repair over the past few decades, there remains a notable gap when compared with other typical two-dimensional materials such as oxides and graphene. It is hoped that through the collaborative efforts of nanotechnology, organic chemistry, and medical researchers, the controllability of BP nanomaterials will continue to improve, superior biological functionalities of BP will be explored, and the cost of BP materials will be reduced. This will ultimately expand their applications in biomedicine and other fields.

## Data Availability

Not applicable.

## Supplementary Materials

The following supporting information can be downloaded at: https://www.mdpi.com/article/10.3390/ph17020242/s1, Figure S1: Peak energy of BP for the different number of layers [12].

## Abbreviations

4D	Four-dimensional
3D	Three-dimensional
2D	Two-dimensional
BP	Black phosphorus
BPNS	Black phosphorus nanosheets
BPQDs	Black Phosphorus quantum dots
NIR	Near infrared
PTT	Photothermal therapy
PDT	Photodynamics therapy
ROS	Reactive oxygen
OH	Oxhydryl
^1^O_2_	Singlet oxygen
ALP	Alkaline phosphatase
HSP	Heat shock protein
PO_4_^3−^	Phosphate
OCN	Osteocalcin
BMP-2	Bone morphogenetic protein-2
Ag	Argentum
Zn	Zinc
SrCL_2_	Strontium chloride
SiO_2_	Silicon dioxide
Ni	Nickel
Ti	Titanium
Mg	Magnesium
VEGF	Vascular endothelial growth factor
bFGF	Basic fibroblast growth factor
β-TCP	β-Tricalcium phosphate
EGCG	Epigallocatechin gallate
4OI	4-Octylitaconic acid
AS	Astragalus IV
DFO	Deferoxamine
PRP	Platelet-rich plasma
Lid	Lidocaine
DOX	Doxorubicin
HA	Hydroxyapatite
Vanco	vancomycin
Apt	Aptamers
Rg1	Ginsenoside Rg1
Hb	Haemoglobin
*E. coli*	*Escherichia coli*
BS	*Bacillus subtilis*
*S. aureus*	*Staphylococcus aureus*
MRSA	Methicillin-resistant *Staphylococcus aureus*
PEA	Polyesteramide
GelMA	Methacrylified gelatin
PVA	Polyvinyl alcohol
CS	Chitosan
Gel	Gelatin
Col	Collagen
HA	Hyaluronic acid
GelMA	Methacrylated gelatin
MSN	Mesoporous silica nanoparticles
PEG	Polyethylene glycol
CNT	Carbon nanotubes
OPF	Fumaric acid
Alg	Sodium alginate
PPy	Polypyrrole
PCL	Polycaprolactone
PLGA	Polylactic acid–glycolic acid copolymer
PLA	Polylactic acid
GO	Graphene
PPF	Polypropylene glycol fumarate
BG	Bioglass
PLLA	Polylactic acid
DLLA-TMC	Poly(lactic acid–co-trimethylene carbonate)
PPS	Polypropylene sulfide
PEDOT	Poly(3,4-ethylenedioxythiophene)
PSS	Polystyrene sulfonate
PAA	Polyacrylic acid
BMSCs	Bone marrow mesenchymal stem cells
HDPSCs	Human pulp stem cells
Saos-2	Osteosarcoma cells
HBMSCs	Human bone marrow mesenchymal stem cells
NIH/3T3	Mouse embryonic cells
MC3T3	Mouse embryonic osteoblasts
MC3T3-E1	Mouse embryonic osteoblast precursor cells
HUVECs	Human umbilical vein endothelial cells
NSCs	Neural stem cells
MSCs	Mesenchymal stem cells
DC	Dendritic cells
MDA-MB-231	Human breast cancer cells
MG -63	Human osteosarcoma cells
hBM-MSCs	human bone marrow mesenchymal stem cells/stromal cells
Nrf2	Nuclear factor E2-like 2
BMP-RUNX2	Bone Morphogenic Protein–runt-related transcription factor 2
PI3K-AKT	Phosphatidylinositide 3 kinases-serine/threonine kinase
HSC	Hematopoietic stem cells
