# Supplementary material for: Advances in the Application of Black Phosphorus-Based Composite Biomedical Materials in the Field of Tissue Engineering
Source: Pharmaceuticals (Basel). 2024 Feb 13;17(2):242. doi: 10.3390/ph17020242 (PMC10892510; doi:10.3390/ph17020242)
Supplement: Supplementary file 1 [file pharmaceuticals-17-00242-s001.zip › pharmaceuticals-2820002-supplementary.pdf]

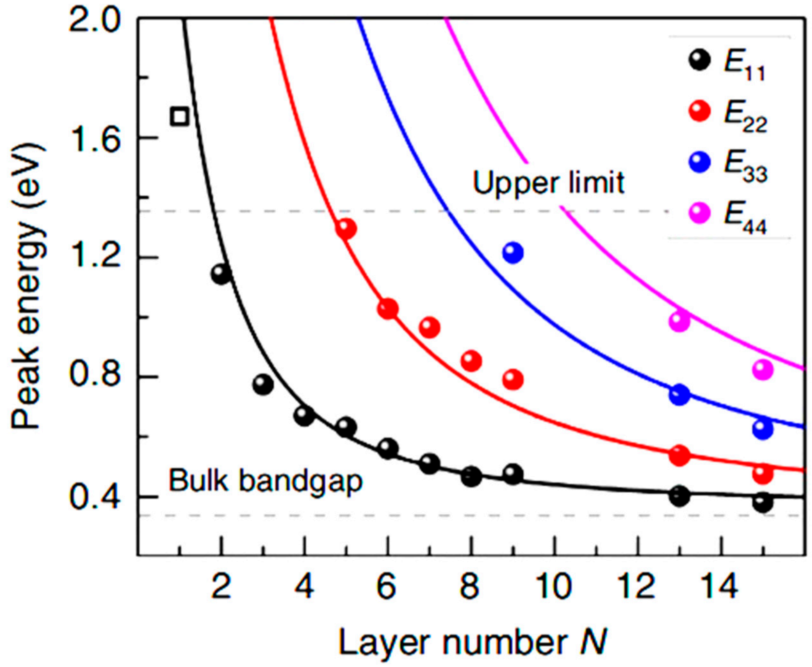

Figure S1 Peak energy of BP for the different number of layers

Figure source:

Zhang G, Huang S, Chaves A, Song C, Özçelik VO, Low T, *et al.* Infrared fingerprints of few-layer black phosphorus. Nat Commun 2017;8:14071. doi: 10.1038/ncomms14071.
